# Organic Reactivity Made Easy and Accurate with Automated Multireference Calculations

**DOI:** 10.1021/acscentsci.3c01559

**Published:** 2024-03-27

**Authors:** Jacob J. Wardzala, Daniel S. King, Lawal Ogunfowora, Brett Savoie, Laura Gagliardi

**Affiliations:** † Department of Chemistry,University of Chicago, Chicago, Illinois 60637, United States; ‡ Davidson School of Chemical Engineering, Purdue University, West Lafayette, Indiana 47906, United States; § Department of Chemistry, Pritzker School of Molecular Engineering, James Franck Institute, Chicago Center for Theoretical Chemistry, University of Chicago, Chicago, Illinois 60637, United States

## Abstract

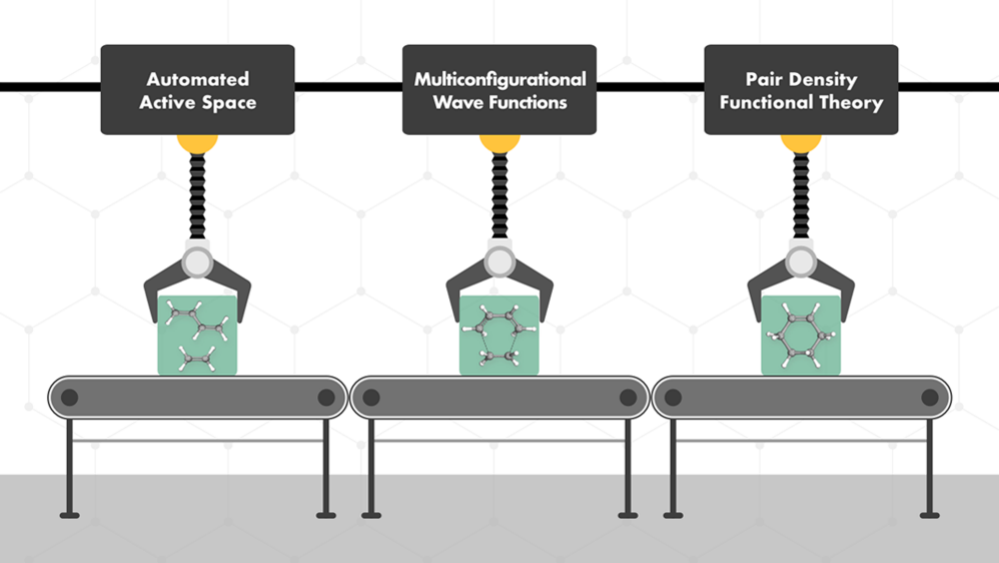

In organic reactivity studies, quantum chemical calculations play a pivotal role as the foundation of understanding and machine learning model development. While prevalent black-box methods like density functional theory (DFT) and coupled-cluster theory (e.g., CCSD(T)) have significantly advanced our understanding of chemical reactivity, they frequently fall short in describing multiconfigurational transition states and intermediates. Achieving a more accurate description necessitates the use of multireference methods. However, these methods have not been used at scale due to their often-faulty predictions without expert input. Here, we overcome this deficiency with automated multiconfigurational pair-density functional theory (MC-PDFT) calculations. We apply this method to 908 automatically generated organic reactions. We find 68% of these reactions present significant multiconfigurational character in which the automated multiconfigurational approach often provides a more accurate and/or efficient description than DFT and CCSD(T). This work presents the first high-throughput application of automated multiconfigurational methods to reactivity, enabled by automated active space selection algorithms and the computation of electronic correlation with MC-PDFT on-top functionals. This approach can be used in a black-box fashion, avoiding significant active space inconsistency error in both single- and multireference cases and providing accurate multiconfigurational descriptions when needed.

## Abstract 2

We introduce an approach that automates accurate calculations of hundreds of organic reactions, based on multiconfigurational pair-density functional theory.

## Introduction

In the past 20 years, quantum chemistry has made great strides in describing chemical reactivity; widely used methods such as density functional theory (DFT) and coupled-cluster methods (e.g., CCSD(T)) have become a rich source of data for the understanding of chemical reactions and the development of machine learning algorithms.^1,2^ However, despite their black-box nature, these methods face limitations on systems poorly described by a single electronic configuration, i.e., multiconfigurational or strongly correlated systems.^3−7^ A key example of these systems is familiar to most chemists: that of the transition state in which the electronic character is often split between describing that of the reactant and of the product. Given the ubiquitous nature of transition states in chemistry, it may then be a wonder how these approaches have proven successful in so many applications. The answer is that for many important cases these methods are simply able to overcome this difficulty despite the fundamental struggle with multiconfigurational character, thanks to favorable error cancellation or a sufficient single reference description. Nevertheless, in automated applications of quantum chemistry such as reaction network exploration,^8^ the poorer description of multiconfigurational species can rear its head in key places and significantly impact results.

As such, describing strong correlation in transition states has long been poised as a potential application for multiconfigurational approaches such as complete active space self-consistent field (CASSCF) theory.^9,10^ This approach overcomes the difficulty of describing multiconfigurational systems by describing the state as a superposition of the possible electronic configurations in an “active space” of orbitals and electrons:^11^
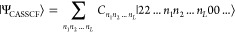
1in which *n*_1_*n*_2_...*n*_*L*_ enumerates the possible occupations of the *L* active orbitals. With a good choice of active space, all static correlation can be addressed with far fewer configurations than FCI and comparable expense to DFT.^12^ However, despite the many academic applications of these approaches in the literature,^13−19^ the widespread adoption of these methods for reactivity has been hindered by the challenge of choosing a consistent and adequate active space along the reaction surface.^20,21^ The CASSCF energy expression is given by
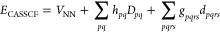
2where *D*_*pq*_ and *d*_*pqrs*_ are the CASSCF one- and two-body reduced density matrices. If the active space is chosen inconsistently between two geometries, one will obtain an unphysical “active space inconsistency error” (ASIE) resulting from the inconsistent treatment of correlation in the density matrices of eq 2. This error generally remains present even when addressing the remaining dynamic correlation perturbatively with methods such as CASPT2^22,23^ or NEVPT2.^24,25^

The most common approach for reducing ASIE involves interpolating the active space orbitals between geometries, providing a continuous set of orbitals along the reaction coordinate.^20^ However, this approach is quite cumbersome: active orbitals often rotate in and out of the active space randomly during this procedure, and the active space may change size along a reaction coordinate, such as when moving from a fairly uncorrelated reactant to a correlated transition state. Furthermore, this interpolation scheme dramatically increases the cost of the calculation relative to approaches such as DFT, as CASSCF calculations are necessary along several points between the reactant and product, whereas KS-DFT only requires calculations at the individual end points.

In this light, we note the broad success of KS-DFT in modeling reactivity, which models all densities via a single determinant and calculates energies via use of an exchange-correlation functional:
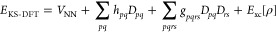
3Despite the fact that the KS-DFT determinant inevitably describes the density matrices of reactants and transition states with different accuracy (i.e., the exact two-body density matrices *d*_*pqrs*_ differ more or less from the single-determinant *D*_*pq*_*D*_*rs*_), KS-DFT is able to obtain good results in reactivity through use of an exchange functional of the density *E*_xc_[ρ]. This statement also applies to the success of “density corrected” DFT (DC-DFT)^26^ in which the densities used in the KS-DFT energy expression (eq 3) come from HF determinants (i.e., the functional has no input on the density, but only the energy calculation). This leads to the hypothesis that the ASIE found in CASSCF and NEVPT2 may come in large part from unequal contribution of the density cumulant between two geometries, *d*_*pqrs*_ – *D*_*pq*_*D*_*rs*_. A multiconfigurational approach that avoids use of the density cumulant by means of an exchange-correlation functional may inherit much of the equal-footing properties of KS-DFT and prove more robust against ASIE.

One such method that achieves this goal is called multiconfigurational pair-density functional theory (MC-PDFT).^27^ This theory more-or-less shares an energy expression with KS-DFT:
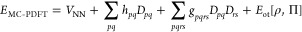
4with two key differences: (i) the exchange-correlation functional is replaced with an “on-top” functional *E*_ot_, which is a functional of both the density ρ and on-top density Π, and (ii) the density arguments *D*_*pq*_, ρ, and Π come from a multiconfigurational (generally CASSCF) wave function. The on-top pair density, derived from the two-particle density matrix, describes the probability of finding two electrons at the same point in space. In practice, the on-top functional is a “translated” functional (most often translated PBE,^28^ tPBE) in which the density and on-top density are used to manufacture effective spin densities for use in the KS-DFT energy expression (eq 3). Thus, as MC-PDFT more-or-less shares eq 3 with KS-DFT, MC-PDFT appears promising for attenuating part of the active space inconsistency error, especially when paired with automated methods for choosing the active space in a reliable and consistent fashion.^12,20,21,29−32^ While MC-PDFT has been tested on a wide variety of systems and excitations,^12,16,33,34^ it has yet to be tested in a high-throughput fashion for reactivity.

Here, we provide the first such test by applying automated MC-PDFT to the calculation of 908 automatically generated organic reactions in the RGD1 database.^35^ These data present a rich variety of organic reactivity and a challenging test for multiconfigurational approaches that is germane to reaction network exploration. Our results highlight the robustness of automated MC-PDFT in this domain compared to other perturbative multiconfigurational approaches such as NEVPT2^24,25^ and outline the opportunity and challenges for applying multiconfigurational methods to high-throughput main-group reactivity. We find that combining the approximate pair coefficient active space selection scheme (APC) with MC-PDFT (referred to as APC-PDFT) generates robust results, with APC-PDFT reproducing DFT results for a set of single reference reactions. In addition, we show the deviation in relative energies from single reference are correlated to the level of multiconfigurational character, with DFT and CCSD(T) becoming less reliable for strongly correlated systems (68% of reactions), and APC-PDFT providing better results in many of these cases.

## Methods

The main barrier to automating multiconfigurational approaches is automatically selecting the active space in a robust fashion. Methods for automatically selecting active spaces continue to be an active research topic, and several approaches exist.^20,21,29−31,34,36−38^ Here, we employ approximate pair coefficient (APC) selection^12,32^ in which candidate Hartree–Fock orbitals are ranked for the active space by means of their approximate pair coefficient interaction with other orbitals. We note that APC is a ranked-orbital approach, where the user defines a maximum active space size. This method allows the practitioner to prevent the selection scheme from picking active spaces larger than are computationally feasible and it also allows for flexibility toward solvers with different practical size limitations (i.e., CAS vs DMRG). The drawback is that the user has to define this maximum size manually, which can result in an unnecessarily large active space. Given doubly occupied orbitals *i* and virtual orbitals *a*, approximate pair coefficients are calculated as
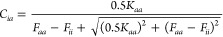
5where *F*_*ii*_, *F*_*aa*_, and *K*_*aa*_ are the respective diagonal elements of the Fock and exchange matrices. The entropies of doubly occupied orbitals *i* and virtual orbitals *a* are then calculated by summing over their approximated interactions (intermediate normalization):
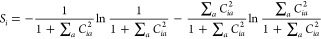
6
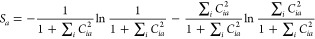
7Interactions with singly occupied orbitals are left uncalculated, and singly occupied orbitals are automatically given the highest possible entropy. As the pair coefficients are generated from Fock and exchange matrix elements, which change adiabatically with the molecular geometry, the APC scheme aims to select moderately consistent (but not exactly consistent) active spaces across the reaction coordinate.

Finally, due to the observed biasing of APC entropies toward doubly occupied orbitals^12,32^ a series of virtual orbital removal steps are employed *N* times in which the highest-entropy virtual orbital is removed from the sums in eqs 6 and 7 and the entropies are recalculated; these highest-entropy virtual orbitals are then assigned the highest entropy at the end of the calculation. For small-to-medium sized organic systems we have found good results with *N* = 2,^12^ which we have used here. However, this parameter appears to have less impact due to the fixed active space size we employ here to enforce active space size consistency between different geometries (described below). Implementation of APC is now available in PySCF.^39,40^

Candidate HF orbitals are then ranked in importance by their orbital entropies, with this ranking used to choose an active space meeting some user-defined size requirement (e.g., a 12 electron in 12 orbital or (12,12) active space). Here, to select consistent active space sizes between geometries, we employ a simple size requirement in which for an (A,B) active space, where A and B are the number of active electrons and orbitals, respectively, the *A*/2 highest-entropy doubly occupied orbitals and the *B* – *A*/2 highest-entropy virtual orbitals are added to the active space; we refer to these active spaces as APC(A,B). CASSCF calculations initialized from these active spaces in the cc-pVDZ basis^41,42^ were then carried out in PySCF.^39,40^ These CASSCF wave functions were then used for the calculation of MC-PDFT (tPBE) and NEVPT2 energies, also implemented in PySCF and PySCF-Forge.^43^

Multiconfigurational (or equivalently, multireference (MR)) character in the resulting wave functions is calculated via the *M*-diagnostic,^44^ which measures multiconfigurational character as a function of the natural orbital occupancies:
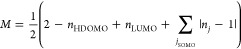
8Here, *n*_HDOMO_, *n*_LUMO_, and *n*_SOMO_ are the average occupations of the highest doubly occupied, lowest unoccupied, and any singly occupied orbitals in the active space. An *M*-diagnostic less than 0.05 is considered minimally multiconfigurational, 0.05 < *M* < 0.1 moderately MR, and *M* > 0.1 substantially MR.

## Data

The reactions for this benchmark were taken from the Reaction Graph Depth 1 (RGD1) data set for CHON-containing molecules.^35^ In brief, these reactions were generated using generic graph-based reaction rules applied to neutral closed-shell reactants sampled from PubChem. Transition state, reactant, and product geometries for each reaction were optimized at the B3LYP-D3/TZVP level. Three subsets of RGD1 were used for this work. These are a random five percent (400) of the break two form one (B2F1) reactions, 400 break two form two (B2F2) reactions, and a “small molecule” data set of 108 reactions in RGD1 with <5 non-hydrogen atoms. The B2F1 reactions, which break two bonds and form one bond as the reaction progresses from reactant to product, have an increased likelihood of showing MR character due to the uneven number of bonds formed and broken in the reaction, whereas the B2F2 reactions, which have two bonds broken and two bonds formed throughout the reaction, have closed-shell reactants and products (Supporting Information). To provide reference results for comparison to the automated multiconfigurational approach, CCSD(T) and B3LYP-D3 (with zero damping) results were recalculated in the cc-pVDZ basis in PySCF using the all-atom preassociated reactants and products provided by RGD1.

## Results

**Figure 1:**
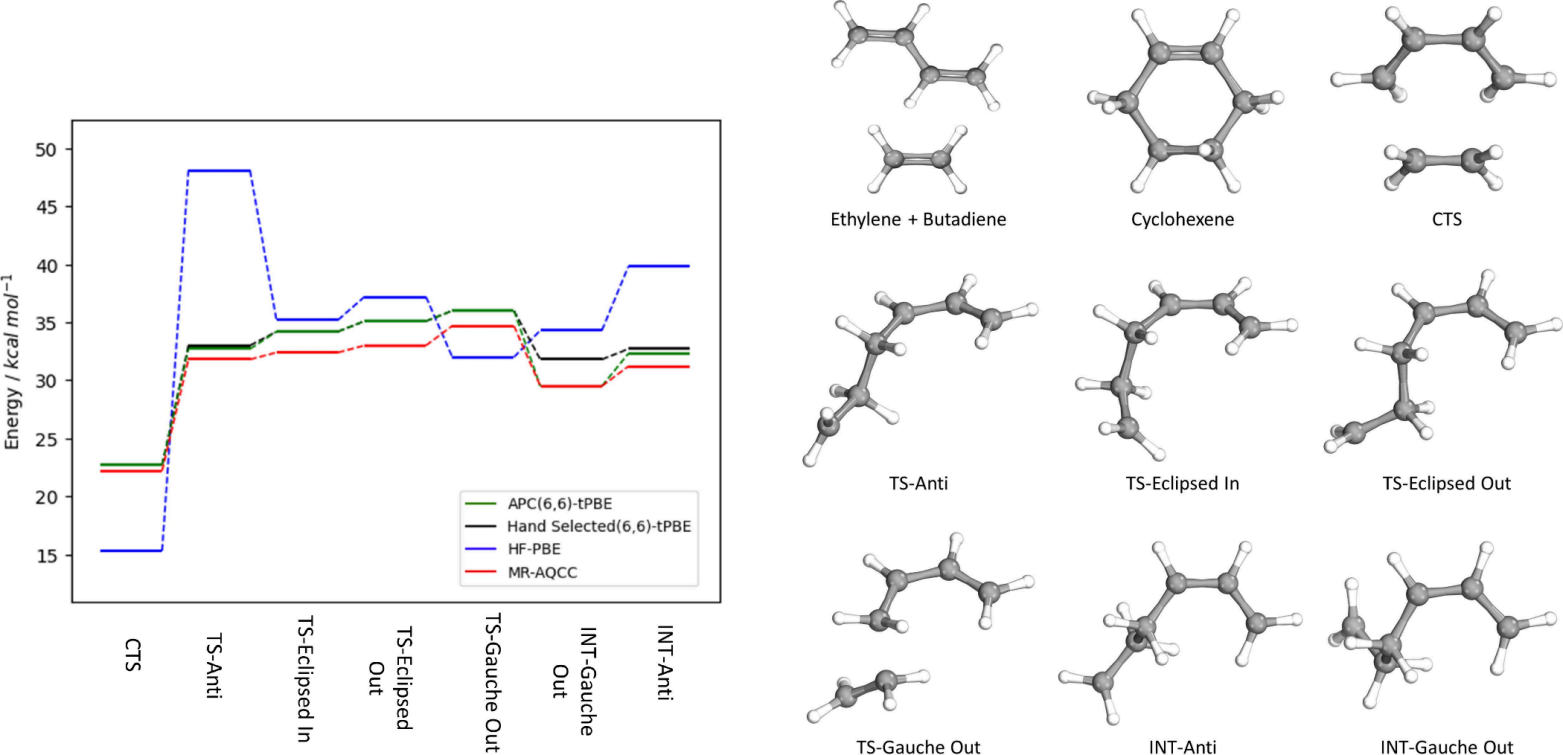
Electronic energies of each state in the concerted transition state (CTS) and biradical reaction pathways relative to the reactants. Four methods are shown: APC(6,6)-tPBE (green, this work), hand-selected (6,6)-tPBE (black),^45^ HF-PBE (blue), and reference MR-AQCC results (red).^46^ The structures of each transition state and intermediate are displayed on the right.

As a first test of our methodology, we explore the performance of APC-tPBE on the Diels–Alder reaction between butadiene and ethylene. This reaction presents a well-studied series of transition states and intermediates^45,46^ that provide a clear challenge for automated multiconfigurational approaches, as all states contain a significant amount of multiconfigurational character (*M* > 0.1). Figure 1 shows the tPBE results obtained with our automated APC(6,6) active spaces compared to previous literature results using hand-selected (6,6) active spaces,^45^ as well as reference multireference averaged quadratic coupled cluster (MR-AQCC) calculations.^46^ The study from Lischka et al. showed the MR-AQCC results to be in good agreement with experiment for the accepted reaction pathway. As is seen, the automatically selected active spaces are able to reproduce the tPBE results (in good agreement with the MR-AQCC results) of the hand-selected active spaces in all transition states, despite not directly enforcing any consistency between active spaces beyond the size. For reference, we show the single-reference limit of MC-PDFT in which the CASSCF wave function densities are replaced with HF densities (equivalent to so-called “density-corrected” PBE^26^); here, we refer to this approach as HF-PBE. Unlike APC(6,6)-tPBE, HF-PBE dramatically overestimates the stability of the concerted transition state (CTS) while greatly underestimating the stability of the TS-Anti transition state and intermediate. Results with an APC(12,12) active space as well as KS-DFT and CCSD(T) are reported in the Supporting Information. The larger active space results are in good agreement with the APC(6,6) performance. Thus, our automated scheme successfully reproduces the important multiconfigurational results.

Given the success of our methodology in reproducing Diels–Alder results, we turn to the 908-reaction subset of RGD1 reactions for further testing. Our calculations show that this set of reactions shows a broad distribution of multiconfigurational character as measured by the *M*-diagnostic (Supporting Information), with 32% of reaction energies and 63% of activation energies demonstrating significant multiconfigurational character (*M* > 0.1), for a total of 68% of reactions exhibiting such character in at least one state overall. To account for the cases with the most multiconfigurational character, we have chosen large APC(12,12) active spaces for each state in these reactions. This active space size is significantly larger than necessary for most reactions in the data set, resulting in inconsistent but unimportant orbitals between the reactants and products of some reactions. These orbital inconsistencies represent a second test of the robustness of MC-PDFT.

**Figure 2:**
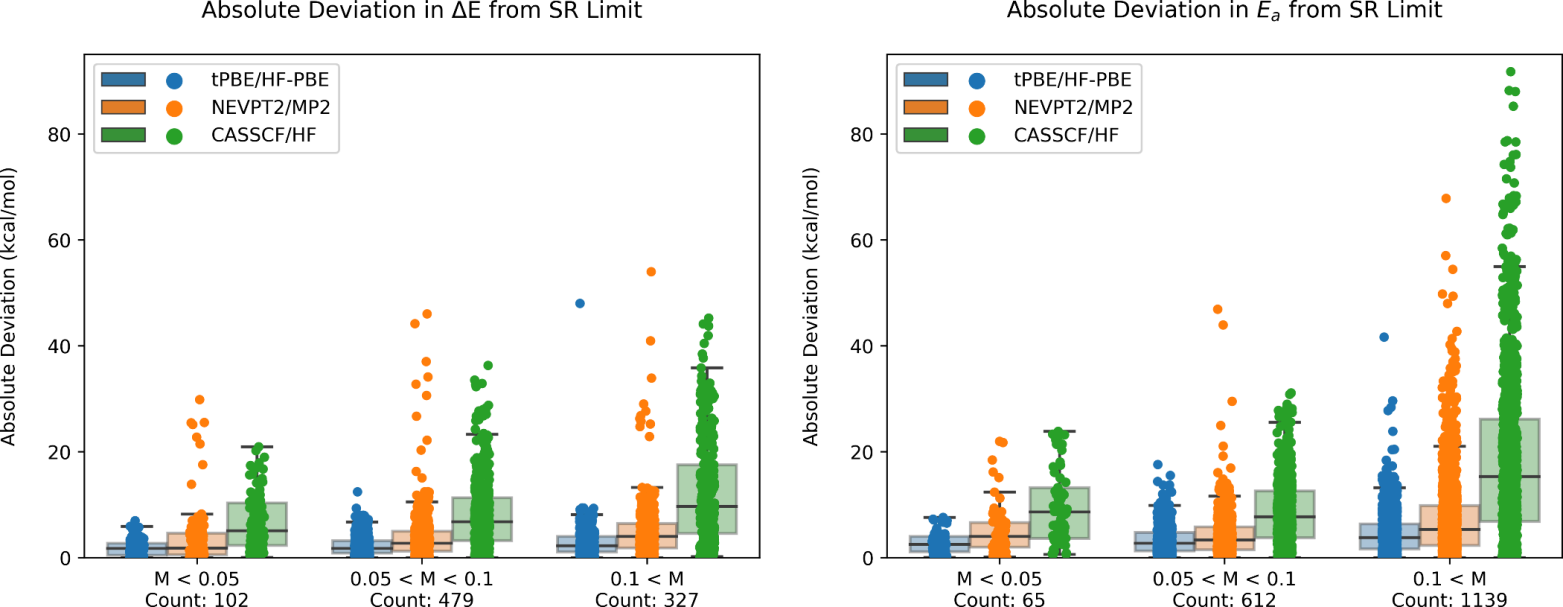
Whisker plots of deviations from single-reference limits (right: Δ*E*; left: *E*_*a*_) of APC-tPBE, APC-NEVPT2, and APC-CASSCF, stratified by the degree of multiconfigurational character as measured by the *M*-diagnostic. The number of reactions in each *M*-diagnostic category are displayed below each label. Mean absolute deviations (MAD) in systems with low multiconfigurational character (*M* < 0.05, in kcal/mol, Δ*E* /*E*_*a*_): 1.8/2.8 (tPBE); 4.0/5.2 (NEVPT2); 6.8/9.8 (CASSCF). Mean absolute deviations (MAD) in systems with high multiconfigurational character (*M* > 0.1, in kcal/mol, Δ*E* /*E*_*a*_): 3.1/4.6 (tPBE); 5.2/7.6 (NEVPT2); 12.3/19.0 (CASSCF).

Figure 2 shows the absolute deviation in the reaction energy, Δ*E*, and the activation energy, *E*_*a*_ (both forward and backward), for all examined reactions from the single reference limit (SRL) for CASSCF (SRL: HF), tPBE (SRL: HF-PBE), and NEVPT2 (SRL: MP2). This deviation is stratified by three degrees of multireference (MR) character: low (*M* < 0.05), 303 moderate (0.05 < *M* < 0.1), and high (0.1 < *M*). As shown clearly, both the mean absolute deviation (MAD) from the SRL and overall spread of the data increases from the low *M* to the high *M* categories. In the cases with low multiconfigurational character, *M* < 0.05, tPBE successfully reproduces the single-reference limit with a mean deviation of ±1.8 kcal/mol for Δ*E* and ±2.8 kcal/mol for *E*_*a*_, with an average between these two of ±2.2 kcal/mol. In contrast, CASSCF and NEVPT2 reproduce these limits with a mean deviation of ±7.9 and 4.4 kcal/mol, respectively, with much larger maximum deviations (as high as 20 kcal/mol). These results show that MC-PDFT is significantly more robust in the single-reference limit toward active space inconsistency error (ASIE) than competing multiconfigurational approaches, making it ideal for high-throughput application. Surprisingly, we find that this robustness carries over to the performance of hybrid PDFT as well, despite it being an admixture of CASSCF and tPBE; this point bears technical discussion and is discussed in the Supporting Information. A similar analysis, using the square of the coefficient of the leading configuration, *C*_0_^2^, as the multireference diagnostic can also be found in the Supporting Information.

**Figure 3:**
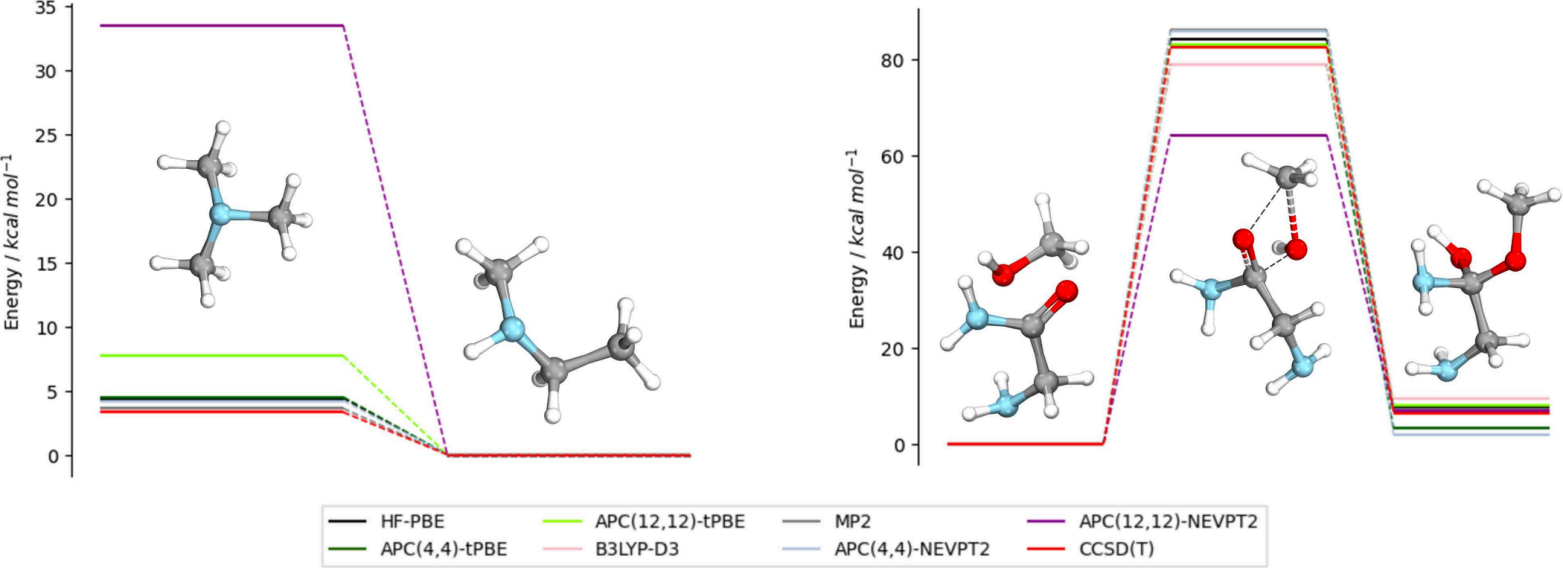
Reactions MR_3361_1 (rearrangement of trimethylamine) and MR_619998_2 (hemiacetal formation from methanol and glycinamide). Six methods are shown on each plot: APC(12,12)-tPBE (light green), APC(12,12)-NEVPT2 (purple), APC(4,4)-tPBE (dark green), APC(4,4)-NEVPT2 (silver), HF-PBE (black), MP2 (gray), B3LYP-D3 (pink), and reference CCSD(T) (red). Energies shown are calculated relative to the lowest energy state (right: reactants; left: products). Since the transition state of the trimethylamine rearrangement reaction is reasonably multireference (*M* = 0.49), it is excluded here.

Two examples where tPBE shows improved reliability for a single-reference reaction are shown in Figure 3. The first is a trimethylamine rearrangement reaction, where the APC(12,12)-CASSCF wave functions for the reactant and product are mostly well-described by a single determinant, with *M*-diagnostics below 0.03. Thus, the overall reaction energy is expected to be similar between each MR approach and its single reference parallel. As is seen, APC-tPBE successfully reproduces HF-PBE to within 3 kcal/mol, a result that is similarly in-line with B3LYP-D3 and CCSD(T). Though this deviation is slightly larger than chemical accuracy, it presents a substantial improvement over APC(12,12)-NEVPT2, which shows a clear deviation from all other methods, overestimating the energy of the reactant by roughly 30 kcal/mol, despite using the same underlying APC-CASSCF wave functions as APC-tPBE. This drastic difference from the single-reference result is emblematic of ASIE, where orbital rotation between the product and reactant results in drastically unphysical results. Since the reaction is known to be single reference, this ASIE can be eliminated through the selection of a smaller active space: APC(4,4)-NEVPT2 produces results in-line with CCSD(T) and density functional approaches, and the APC(4,4)-tPBE results come closer in-line with CCSD(T).

The second case presents the formation of a hemiacetal from methanol and glycinamide. Here, all three states exhibit an *M* of less than 0.05, indicating both the reaction and activation energies should be well described by a single-determinant wave function. Despite this, both the forward and reverse barriers are predicted to be 20 kcal/mol lower with APC(12,12)-NEVPT2 than MP2. By comparison, APC-tPBE agrees to within chemical accuracy (1 kcal/mol) with the single-reference limit of HF-PBE and CCSD(T). Once again, the smaller APC(4,4) active space largely remedies this unphysical error with NEVPT2, demonstrating the error to be due to ASIE. An in-depth evaluation of the active space dependence of tPBE and NEVPT2 for these two reactions, as well as CASSCF, is included in the Supporting Information.

**Figure 4:**
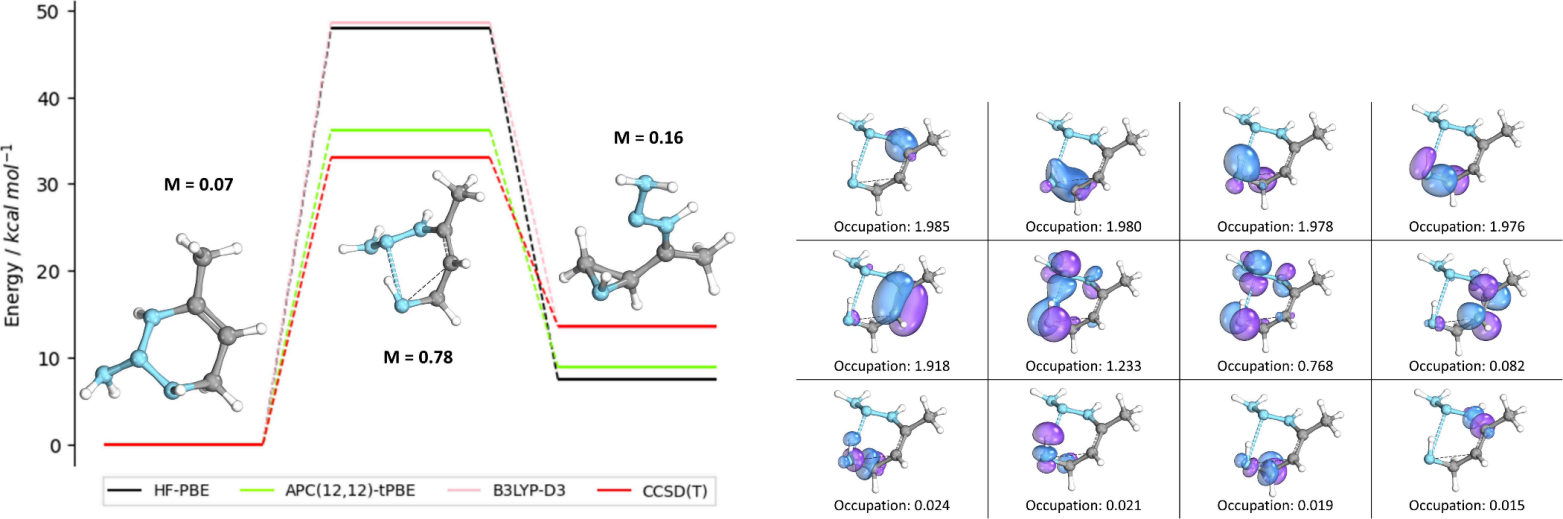
Reaction MR_186317_0 (ring-opening/ring-closing reaction of N_4_C_4_H_10_). The APC(12,12)-tPBE (green), HF-PBE (black), B3LYP-D3 (pink), and CCSD(T) (red) energy diagrams are displayed on the left. The transition state active orbitals and their occupations are shown on the right.

We next show by example how multiconfigurational effects can result in important deviations from DFT and CCSD(T) in the RGD1 data set. The first example is shown in Figure 4, which highlights the most common type of deviation from single reference in which the transition state exhibits the largest degree of multiconfigurational character (*M* = 0.767). The transition state orbitals of this ring-opening/ring-closing reaction show significant multiconfigurational character in both the bond breaking of the 6-membered ring and the C–C double bond rearranging to form the 3-membered ring. The concerted nature of this ring-opening reaction makes this a difficult case for single-reference approaches, much like the Diels–Alder reaction studied prior (Figure 1). As a result, B3LYP-D3 and HF-PBE overestimate the activation energy of the forward reaction by 12 kcal/mol relative to APC-tPBE. In this case, the multiconfigurational character is able to be captured by CCSD(T), which is largely in agreement with the automated APC-tPBE results. The chosen orbitals and their occupations for the transition state are shown alongside the energy diagram.

**Figure 5:**
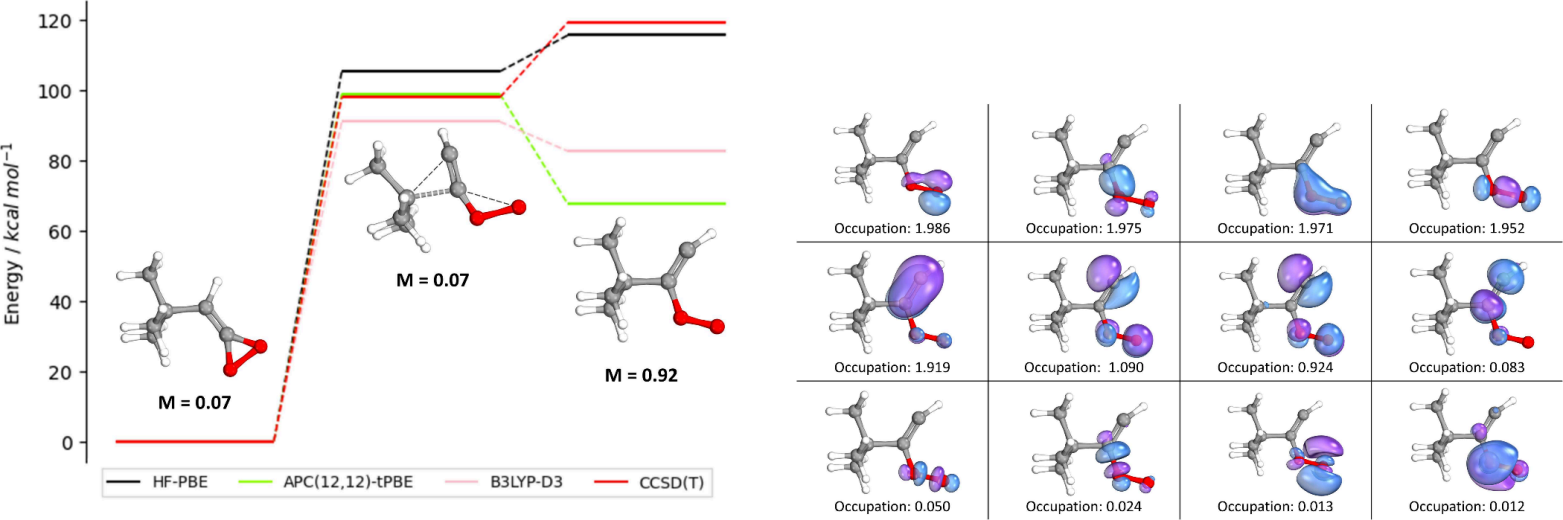
Reaction MR_673407_0 (ring opening of 3-membered heterocycle). The APC(12,12)-tPBE (green), HF-PBE (black), B3LYP-D3 (pink), and CCSD(T) (red) energy diagrams are displayed. The product active orbitals and their occupations are shown on the right.

Figure 5 presents a second case in which the ring opening of a 3-membered heterocycle forms an oxygen diradical with significant multiconfigurational character. As is seen, the HF determinant is completely incapable of describing this diradical product, overestimating the energy of this product relative to the reactant by 60 kcal/mol—higher in energy than the transition state. Due to this terrible description given by HF, CCSD(T) also dramatically overestimates the energy of the biradical relative to the transition state. The unrestricted nature of B3LYP-D3 is able to account for the multiconfigurational character of the biradical somewhat, predicting a shallow barrier of 8.5 kcal/mol relative to the transition state. In contrast, APC-tPBE predicts a significantly more stable product, with a barrier of 31.3 kcal/mol relative to the transition state, and in much better agreement with the CCSD(T) reference values for the single-reference reactant and transition state. We believe these APC-tPBE results give a much more accurate description than either DFT or CCSD(T), and serve to highlight the necessity of multiconfigurational approaches for some reactions containing significant multiconfigurational character.

As a study of basis set dependence, we have investigated the behavior of B3LYP, APC-tPBE, and CCSD(T) in the larger cc-pVTZ basis for the case studies presented in Figures 3–5 (Supporting Information). Overall, we find the APC-tPBE to be remarkably consistent with respect to basis set size, with nearly all results in the cc-pVDZ basis set being well-reproduced in the larger cc-pVTZ basis and qualitatively similar correlating orbitals being chosen in all cases. However, a large discrepancy is found in the cc-pVTZ description of MR_673407_0 in which the APC(12,12)-tPBE reaction energy changes from 31.3 kcal/mol in the cc-pVDZ basis to 12.9 kcal/mol in the cc-pVTZ basis. We find that this discrepancy is due to an abnormally large ASIE in the cc-pVTZ basis, which can be eliminated by executing a CASCI in only the (4,4) active space of correlating orbitals (visually identical to those of the cc-pVDZ basis), which largely reproduces the results shown in Figure 5. This process of recomputing reaction energies using CASCI calculations in only the space of correlating orbitals is promising for further reducing ASIE in APC-tPBE and will be explored in future work.

## Discussion/Conclusion

We have here presented the first large-scale automated multiconfigurational approach to the modeling of organic reactivity, which provides a compelling alternative to DFT and CCSD(T) for interrogating chemical space. These multiconfigurational methods have been held back from high-throughput application for decades due to the problem of active space inconsistency error (ASIE), which is here overcome through the increased robustness of the MC-PDFT method to ASIE and automated active space selection with the approximate pair coefficient (APC) approach. We have applied this automated APC-PDFT approach to the calculation of 908 main group reactions from the RGD1 database, which successfully reproduces the single-reference limit with ASIE of ±2.2 kcal/mol (similar to deviations between different density functionals) while providing more accurate multiconfigurational descriptions than DFT and CCSD(T) in many of the 68% of reactions containing multiconfigurational character. Taken at face value, these results make it possible for the first time to envision the high-throughput use of multiconfigurational methods in this domain, potentially increasing the accuracy of predictions at significantly lower cost (and possibly higher accuracy) than CCSD(T).

Of course, there are limitations. First, there is no reason to expect good results if a sufficient active space is not chosen for all geometries. In the best case, one will reproduce HF-PBE, which may or may not be adequate.^47^ In the worst case, describing only some multiconfigurational states with good active spaces may result in an imbalanced treatment and actively worse predictions. How can one be sure that this is not the case? The APC(12,12) active spaces chosen in this work seem to have been sufficient for this application, but further development will be needed for application to larger organic complexes and transition metal systems. Ultimately, different approaches need to be tested on a wide variety of systems and investigated on a case-by-case basis to be trusted.

Second, the active space dependence of MC-PDFT may be larger than is comfortable in some sensitive systems. For example, previous work on H_2_ dissociation has shown that the predicted dissociation energy of MC-PDFT can vary by over 10 kcal/mol, increasing the active space size from a minimal (2,2) to (2,28).^48^ Nevertheless, this work has shown that cases such as this are more likely to be outliers than the norm; H_2_ dissociation is a well-known failing of restricted HF and DFT, and thus the active space likely has an outsized impact on the performance of MC-PDFT in this case. The generally active-space-independent nature of APC-PDFT beyond a minimum size is further shown by recent studies calculating vertical excitation energies.^12^

Regardless of these remaining challenges, the throughput, automation, and robustness achieved here represent a milestone in applying multiconfigurational methods to main group reactivity and suggest further general-use implementations are possible. The next frontier involves extending this approach to encompass full reaction networks and larger compounds, promising a more comprehensive understanding of complex chemical processes.

## Data Availability

Converged CI vectors, molecular orbital coefficients, and energies for all reactions can be found at: 10.5281/zenodo.10265717.

## Supporting Information Available

Full energetics for the Diels–Alder reaction, plots of signed deviations for CASSCF, tPBE, tPBE0, and NEVPT2, discussion of other multiconfigurational diagnostics, and active space dependence of case studies (PDF)
Transparent Peer Review report available (PDF)
