# Supplementary material for: Organic Reactivity Made Easy and Accurate with Automated Multireference Calculations
Source: ACS Cent Sci. 2024 Mar 27;10(4):833–41. doi: 10.1021/acscentsci.3c01559 (PMC11046455; doi:10.1021/acscentsci.3c01559)
Supplement: Supplementary file 1 — oc3c01559_si_001.pdf [file oc3c01559_si_001.pdf]

Supporting Information: Organic Reactivity Made Easy and Accurate with Automated Multireference Calculations

Jacob J. Wardzala,† Daniel S. King,† Lawal Ogunfowora,‡ Brett Savoie,‡ and Laura Gagliardi*,¶

†*Department of Chemistry, University of Chicago, Chicago, IL 60637*

‡*Davidson School of Chemical Engineering, Purdue University, West Lafayette, IN 47906*

¶*Department of Chemistry, Pritzker School of Molecular Engineering, James Franck Institute, Chicago Center for Theoretical Chemistry, University of Chicago, Chicago IL 60637*

E-mail: lgagliardi@uchicago.edu

Contents

1 Diels-Alder Reaction **S-2**

1.1 Energetics S-2

1.2 M Diagnostic S-3

2 RGD1 Database **S-3**

2.1 Distribution of Multireference Character in the RGD1 Database S-4

2.2 Absolute and Signed Deviations from the Single Reference Limit S-5

2.3 Discussion of Hybrid PDFT S-6

2.4 Absolute and Signed Deviations from B3LYP-D3 . . . . S-8
2.5 C_0^2 as a Multiconfigurational Diagnostic: Absolute and Signed Deviations from the Single Reference Limit S-9
2.6 Discussion on the Robustness of MC-PDFT Towards ASIE S-11
2.7 Case Studies: MR_3361_1 . . . . S-12
2.8 Case Studies: MR_619998_2 . . . . S-15
2.9 Case Studies: MR_186317_0 . . . . S-18
2.10 Case Studies: MR_673407_0 . . . . S-20

References **S-24**

1 Diels-Alder Reaction

We present the reproduction of the hand-selected Diels-Alder results by the APC(12,12) method applied to the RGD1 dataset.

1.1 Energetics

Table S1: Energies of each state in the CTS and biradical reaction pathways relative to the reactants. APC(6,6), APC(12,12), and hand selected(6,6)[S1] results refer to CASSCF-tPBE energies. MR-AQCC was also calculated with reference to a CASSCF(6,6) wavefunction.[S2] The APC(6,6) results reproduce the hand-selected results nearly exactly, while the APC(12,12) results reproduce the hand-selected results with only minor differences.

	ΔE (kcal/mol)				
State	MR-AQCC	APC(6,6)	APC(12,12)	Hand Selected(6,6)	HF-PBE
CTS	22.2	22.7	24.3	22.7	15.3
TS-anti	31.8	32.7	35.7	33.0	48.1
TS-eclipsed-in	32.4	34.2	36.5	34.2	35.2
TS-eclipsed-out	33.0	35.1	37.4	35.1	37.2
TS-gauche-out	34.7	36.0	37.8	36.0	32.0
INT-anti	29.5	29.5	33.8	31.8	34.3
INT-gauche-out	31.2	32.3	35.3	32.7	39.8

Table S2: Energies of each state in the CTS and biradical reaction pathways relative to the reactants using PBE-D3, B3LYP-D3 and CCSD(T).

	ΔE (kcal/mol)		
State	PBE-D3	B3LYP-D3	CCSD(T)
CTS	12.1	18.7	21.5
TS-anti	27.5	30.2	29.8
TS-eclipsed-in	34.4	48.0	35.7
TS-eclipsed-out	36.4	50.4	36.5
TS-gauche-out	29.5	40.8	38.3
INT-anti	33.3	47.1	32.8
INT-gauche-out	38.1	51.6	33.2

1.2 M Diagnostic

Table S3: M Diagnostic for each state examined in the two Diels-Alder reaction pathways. M diagnostics are shown with each active space selection method: hand selected(6,6)[S1] and APC(6,6) and APC(12,12). The APC M-diagnostics reproduce those of the hand-selected active spaces in all cases.

State	M Hand Selected(6,6)	M APC(6,6)	M APC(12,12)
1,3-Butadiene	0.118	0.118	0.120
Ethylene	0.088	0.088	0.082
CTS	0.135	0.135	0.125
TS-anti	0.994	0.993	0.992
TS-eclipsed-in	0.775	0.775	0.764
TS-eclipsed-out	0.808	0.808	0.797
TS-gauche-out	0.584	0.584	0.558
INT-anti	0.803	0.801	0.791
INT-gauche-out	0.862	0.858	0.855

2 RGD1 Database

We discuss the distribution of multiconfigurational character between the different datasets. We then show the absolute and signed deviations from the single-reference limits of each methods. We also discuss the surprising robustness of tPBE0 towards active space inconsistency error (ASIE), the use of C_0^2 as a multireference diagnostic, and give more detail about the case studies.

2.1 Distribution of Multireference Character in the RGD1 Database

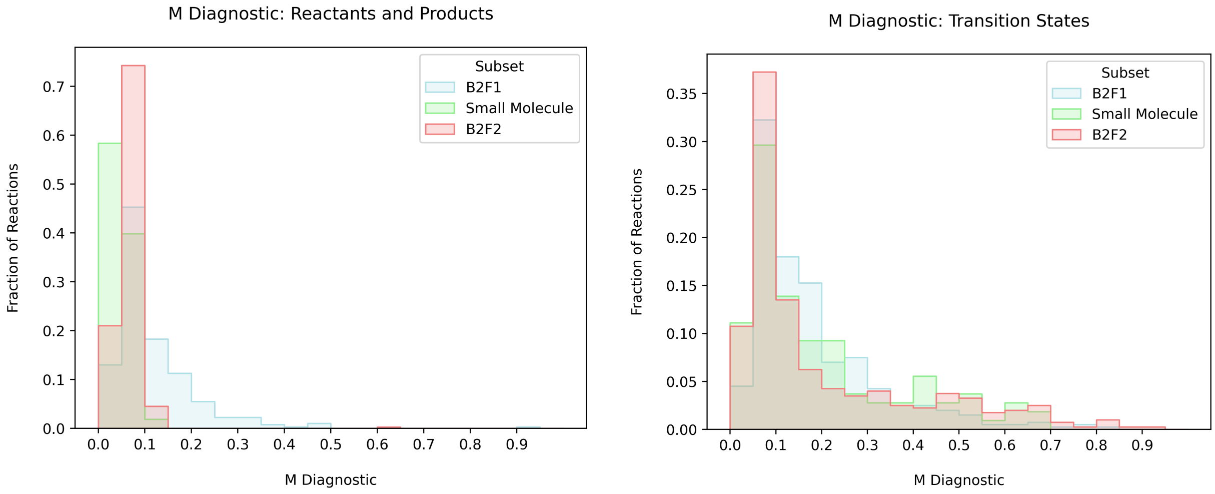

Figure S1: Distribution of MR character, defined by the M diagnostic, in the reactant and product states (left) and the transition states (right).

Figure S1 shows the calculated distributions of multiconfigurational character found with the automated approach in the three subsets of data from RGD1, for both the energy difference between the reactants and products (ΔE) and the energy difference between the end points and the transition states (E_a). The ΔE results provide an intuitive distribution of multiconfigurational character, with the dataset of small systems having significantly smaller multiconfigurational character than the B2F2 and B2F1 datasets, due to the loss of ground-state multiconfigurational nature such as aromaticity. Likewise, the B2F1 dataset has a significantly larger tail of multiconfigurational systems due to a number of reactants and products with unstable binding conformations. Further in line with one's intuitive understanding of multiconfigurational character is the much larger proportion of transition states that exhibit significant static correlation than the products or reactants. However, the distribution of multiconfigurational character across the subsets of data is very similar in the transition states. Consistent with the picture of all bond dissociations engendering some multiconfigurational character, all subsets of data have long tails of reactions with large

M-diagnostics.

2.2 Absolute and Signed Deviations from the Single Reference Limit

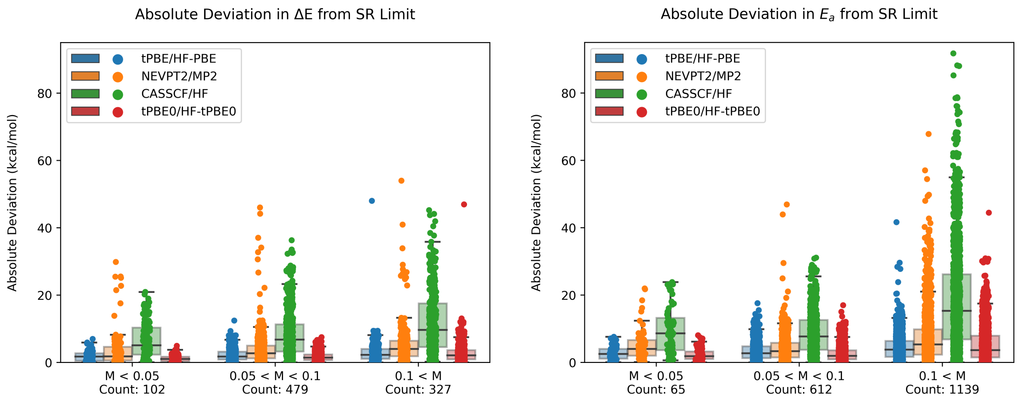

Figure S2: Whisker plots of absolute deviations from single reference limits (right: E_a, left: ΔE) of APC-tPBE, APC-NEVPT2, APC-CASSCF, and APC-tPBE0, stratified by the M diagnostic. Surprisingly, we find that tPBE0 demonstrates similar robustness compared to tPBE against ASIE in the weakly correlated systems ($M < 0.05$).

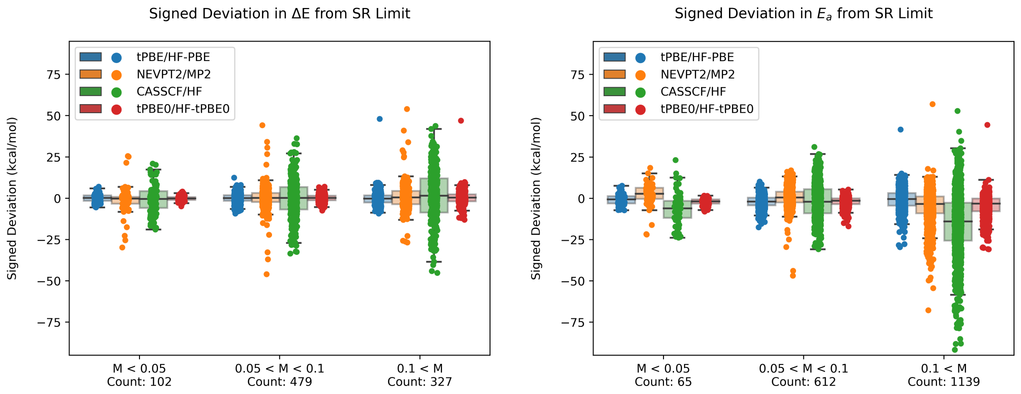

Figure S3: Whisker plots of signed deviations from single reference limits (right: E_a, left: ΔE) of APC-tPBE, APC-NEVPT2, APC-CASSCF, and APC-tPBE0, stratified by the M diagnostic. No systematic error is observed except for the somewhat systematic underestimation of CASSCF in the multireference cases ($M > 0.1$), due to the larger amount of static correlation captured by CASSCF in the transition state.

2.3 Discussion of Hybrid PDFT

The hybrid PDFT energy expression is given by a weighted average of the CASSCF energy and the tPBE energy:[S3]

$$E_{\text{HMC-PDFT}} = (1 - X)E_{\text{MC-PDFT}} + XE_{\text{CASSCF}} \tag{1}$$

For tPBE0, we define $X = 0.25$, equivalent to the percent of exchange mixing in PBE0.[S4] Thus the single reference limit is given by

$$E_{\text{HF-tPBE0}} = (1 - X)E_{\text{HF-PBE}} + XE_{\text{HF}} \tag{2}$$

and the deviation from the single-reference limit is given as a weighted average of the SRL deviations of tPBE and CASSCF:

$$\frac{3}{4}(\Delta E_{\text{tPBE}} - \Delta E_{\text{HF-PBE}}) + \frac{1}{4}(\Delta E_{\text{CASSCF}} - \Delta E_{\text{HF}}) \tag{3}$$

Because of this, it would well be expected that the robustness of tPBE0 in the single-reference limit would be diminished by the poor performance of CASSCF shown in figure S2. However, this is not the case – surprisingly, we find that tPBE0 is slightly more robust towards ASIE than tPBE with mean absolute deviations of 1.1/2.4 kcal/mol for $M < 0.05$ for $\Delta E/E_a$, respectively.

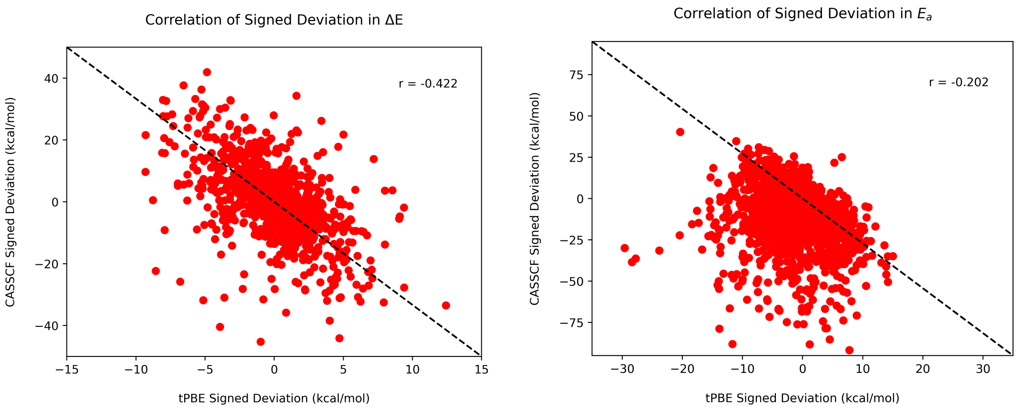

Figure S4: Correlation plots (right: E_a, left: ΔE) of CASSCF signed error (relative to HF) and tPBE signed error (relative to HF-PBE). The correlation coefficient is given for each plot. Pearson's r = -0.42 and -0.20 for ΔE and E_a respectively.

The only explanation is that the deviations from the SRL for tPBE and CASSCF are inversely correlated, resulting in an unexpectedly robust method against ASIE. The correlation between the signed ASIE of tPBE and signed ASIE of CASSCF (those shown in Figure S3) is given in Figure S4. For ΔE, the inverse correlation is particularly clear, though a weaker correlation remains for E_a. This correlation produces favorable error cancellation and thus on average slightly smaller deviations for tPBE0 from the SRL.

2.4 Absolute and Signed Deviations from B3LYP-D3

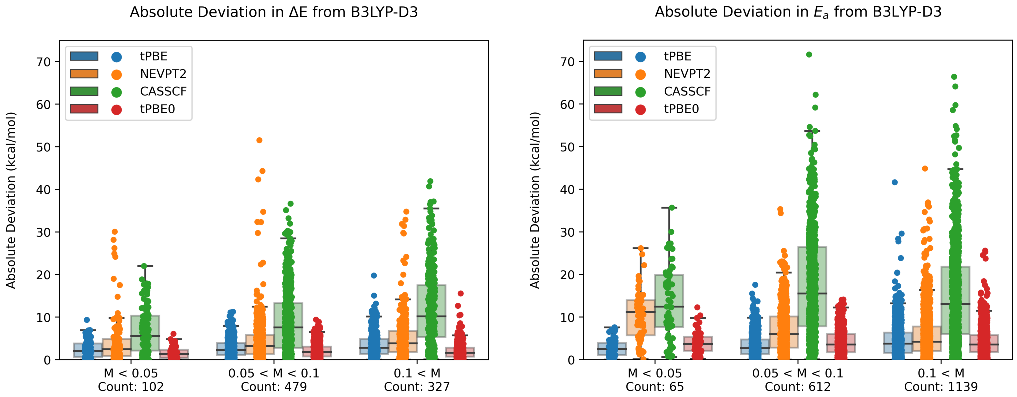

Figure S5: Whisker plots of absolute deviations from B3LYP-D3 (right: E_a, left: ΔE) of APC-tPBE, APC-NEVPT2, APC-CASSCF, and APC-tPBE0, stratified by the M diagnostic. Mean absolute deviations (MAD) in systems with low multiconfigurational character ($M < 0.05$, in kcal/mol, ΔE /E_a): 2.4/2.9 (tPBE); 4.4/10.3 (NEVPT2); 6.8/13.4 (CASSCF); 1.5/4.0 (tPBE0). Mean absolute deviations (MAD) in systems with high multiconfigurational character ($M > 0.1$): 3.6/4.5 (tPBE); 5.1/5.9 (NEVPT2); 11.8/15.0 (CASSCF); 2.1/4.1 (tPBE0). Similar robustness is observed compared to the deviations from the single-reference limit (Figure S2).

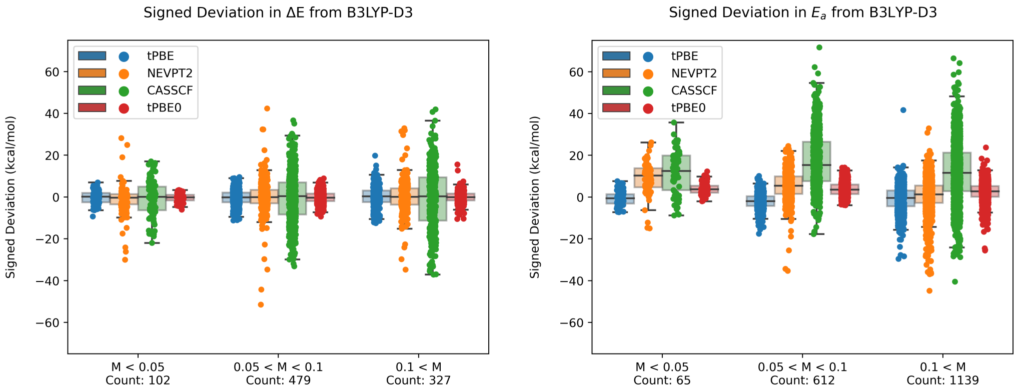

Figure S6: Whisker plots of signed deviations from B3LYP-D3 (right: E_a, left: ΔE) of APC-tPBE, APC-NEVPT2, APC-CASSCF, and APC-tPBE0, stratified by the M diagnostic. CASSCF is seen to routinely overestimate activation energies compared to B3LYP-D3.

2.5 C_0^2 as a Multiconfigurational Diagnostic: Absolute and Signed Deviations from the Single Reference Limit

To further support this work, and specifically the role of the M diagnostic as a tool for describing the multiconfigurational character of a system, we also performed the same analysis of reaction and activation energy deviations (absolute deviations: Figure S7, signed deviations: Figure S8) from the single reference limit using the square of the CI coefficient of the leading configuration in the CASSCF wavefunction (C_0^2). $C_0^2 < 0.90$ indicates a significantly multiconfigurational system. An intermediate category, $0.925 > C_0^2 > 0.90$, is also used to indicate a somewhat multiconfigurational character. C_0^2 is calculated in the natural orbital basis. As with the M diagnostic, we see that NEVPT2 is less robust than tPBE/tPBE0 in reproducing the single reference energies for the low multiconfigurational character reactions (those where $C_0^2 > 0.925$). We also see the same expected increased deviations for the multiconfigurational reactions ($C_0^2 < 0.90$). The squared leading coefficient is shown to be strongly correlated with the M diagnostic in Figure S9.

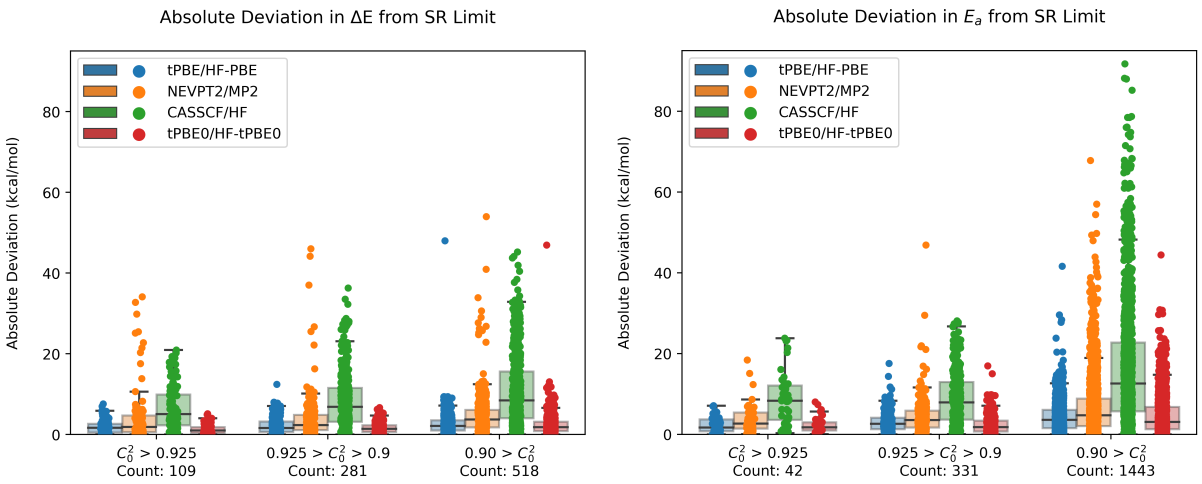

Figure S7: Whisker plots of deviations from single-reference limits (right: ΔE left: E_a) of APC-tPBE, APC-NEVPT2 and APC-CASSCF, stratified by the degree of multiconfigurational character as measured by C_0^2. The number of reactions in each M diagnostic category are displayed below each label. Mean absolute deviations (MAD) in systems with low multiconfigurational character ($C_0^2 > 0.925$, in kcal/mol, ΔE /E_a): 1.9/2.3 (tPBE); 4.4/4.1 (NEVPT2); 6.7/8.9 (CASSCF). Mean absolute deviations (MAD) in systems with high multiconfigurational character ($C_0^2 < 0.90$, in kcal/mol, ΔE /E_a): 2.6/4.3 (tPBE); 4.7/6.9 (NEVPT2); 10.9/16.8 (CASSCF).

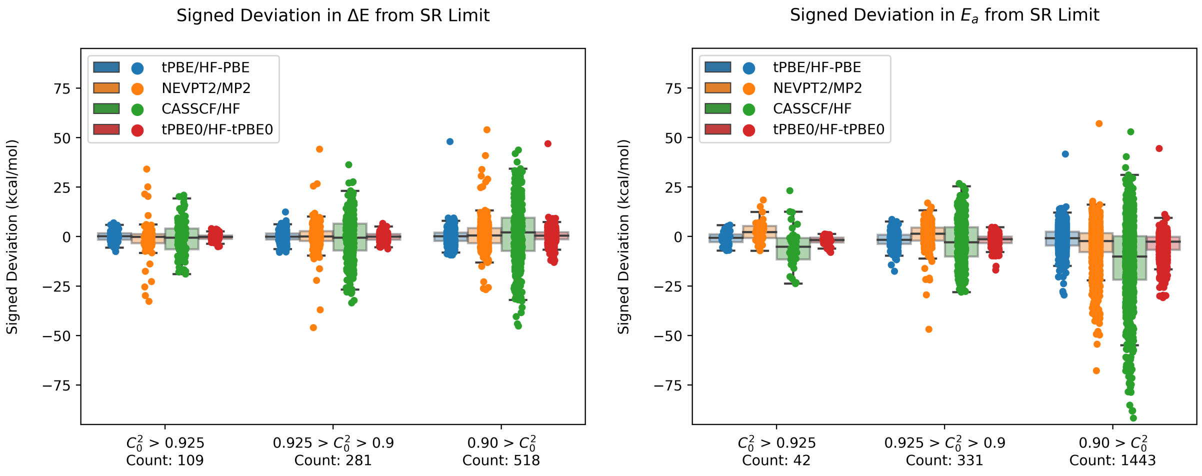

Figure S8: Whisker plots of signed deviations from single-reference limits (right: ΔE left: E_a) of APC-tPBE, APC-NEVPT2 and APC-CASSCF, stratified by C_0^2.

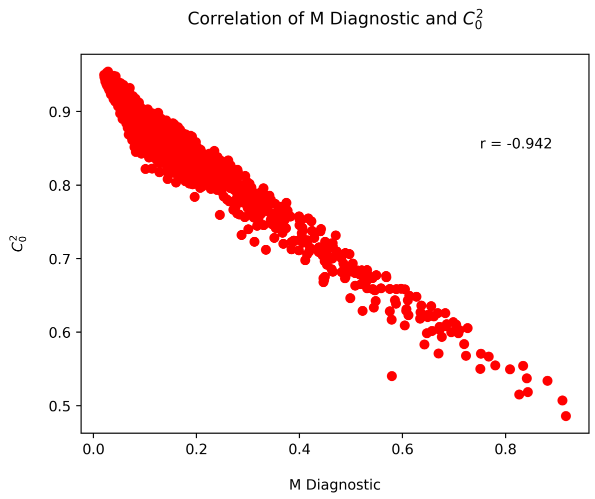

Figure S9: Correlation of the M diagnostic and C_0^2 across the RGD1 dataset. Pearson's r = -0.94.

2.6 Discussion on the Robustness of MC-PDFT Towards ASIE

As shown in several examples throughout the manuscript and supporting information, MC-PDFT appears to have a dramatically increased robustness towards ASIE as compared to CASSCF and NEVPT2. The theoretical reasoning for why this is the case is worth discussing in greater detail here. As presented in the introduction, we hypothesize that the greater ASIE of CASSCF and NEVPT2 is due to the unequal energetic contributions of inconsistent orbitals to the 2-RDM between geometries. As MC-PDFT excludes the 2-RDM from its energy expression (except indirectly through the on-top density Π), it is significantly more robust to these orbital inconsistencies.

Additionally, NEVPT2 can often be more prone to ASIE than CASSCF (as shown for MR_3361_1 below) as the perturber states also change as a function of the active orbitals. While it is true that NEVPT2 should converge appropriately in the FCI limit,making the perturbative treatment redundant, the CAS active space size is very far from this limit even for the small-to-medium-sized molecules studied here. For example, the molecules in Figure 3 contain a total of 25 and 38 valence orbitals, respectively, significantly larger than the (12,12) space treated multiconfigurationally. As such, larger APC active spaces can

often perform much worse with these active spaces due to ASIE. If larger active spaces are imbalanced or not converged, significant changes in NEVPT2 results relative to smaller, balanced active spaces can occur, highlighting the strong dependence of NEVPT2 on the active space. Comparatively, MC-PDFT is more robust to the active space choice, making it an advantageous method for multireference reactivity calculations.

Below, we provide studies of the ASIE in CASSCF, NEVPT2, and tPBE with increasing active space size for the reactions shown in Figure 3, as well as orbitals and cc-pVTZ results for all case studies shown in the manuscript.

2.7 Case Studies: MR_3361_1

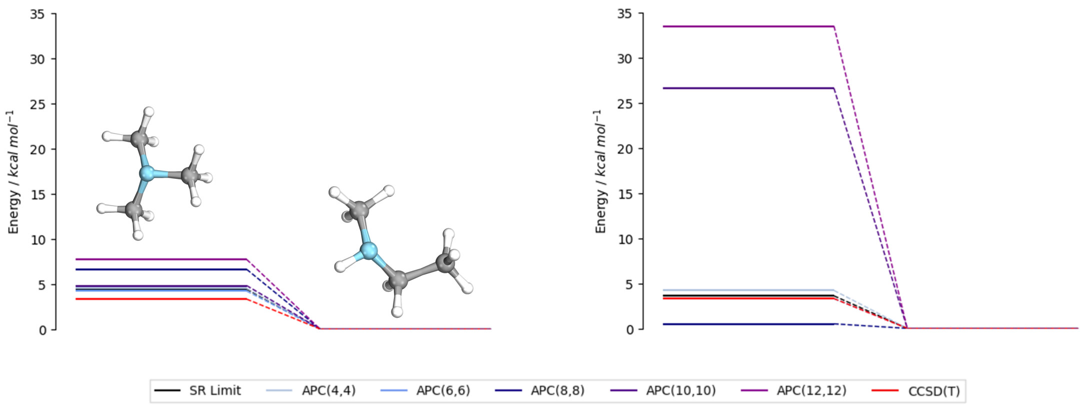

Figure S10: Reaction MR_3361_1 (rearrangement of trimethylamine) with the cc-pVDZ basis. Active space dependence of tPBE (left) and NEVPT2 (right) shown with HF-PBE/MP2, APC(4,4), APC(6,6), APC(8,8), APC(10,10), and APC(12,12) results with each method. CCSD(T) is included as a reliable reference for single reference reactions. Energies are calculated relative to the lowest energy state. Since the transition state of this reaction is reasonably multireference, it is excluded in this discussion.

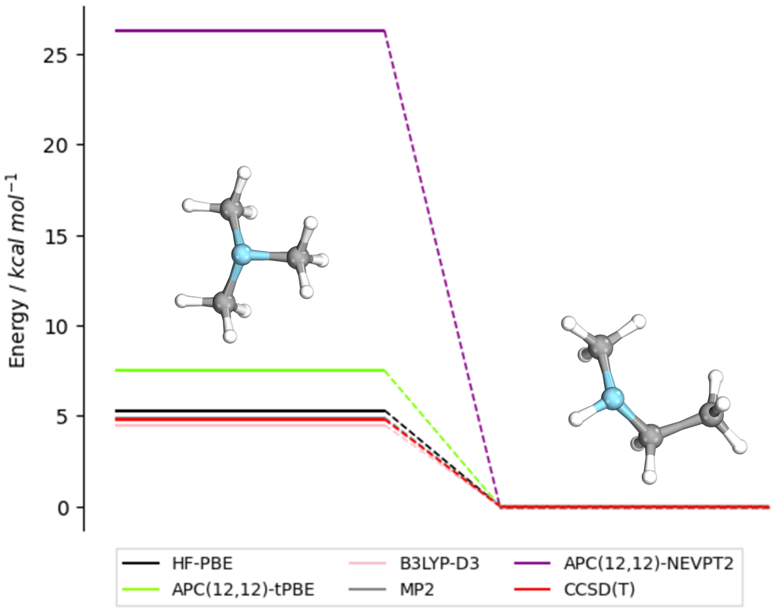

Figure S11: Reaction MR_3361_1 (rearrangement of trimethylamine) with the larger cc-pVTZ basis. The APC(12,12)-tPBE (green), APC(12,12)-NEVPT2 (purple), MP2 (grey), HF-PBE (black), B3LYP-D3 (pink), and CCSD(T) (red) energy diagrams are displayed on the left.

Table S4: Reaction energies for reaction MR_3361_1 as a function of APC active space size with the cc-pVDZ basis. The NEVPT2 result is shown to converge to the correct single-reference limit as the active space size is decreased.

Active Space	Method	ΔE
SR	HF-PBE	-4.4
	MP2	-3.6
	B3LYP-D3	-3.4
	CCSD(T)	-3.3
APC(12,12)	tPBE	-6.7
	NEVPT2	-33.5
APC(10,10)	tPBE	-5.9
	NEVPT2	-26.6
APC(8,8)	tPBE	-6.6
	NEVPT2	-0.5
APC(6,6)	tPBE	-4.2
	NEVPT2	-3.3
APC(4,4)	tPBE	-4.5
	NEVPT2	-4.2

Table S5: Reaction energies for reaction MR_3361_1 with the larger cc-pVTZ basis set.

Active Space	Method	ΔE
SR	HF-PBE	-5.3
	MP2	-4.9
	B3LYP-D3	-4.5
	CCSD(T)	-4.8
APC(12,12)	tPBE	-7.5
	NEVPT2	-26.3

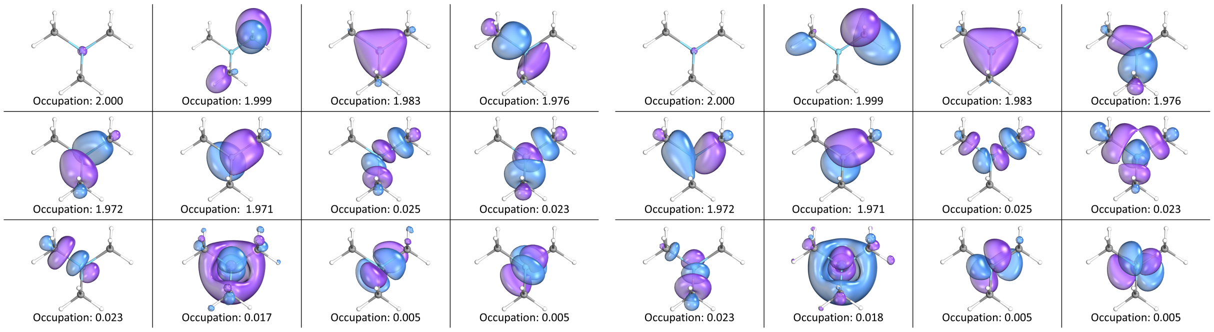

Figure S12: APC(12,12) selected active orbitals for the reactant in reaction MR_3361_1 with the cc-pVDZ (left) and cc-pVTZ (right) basis sets.

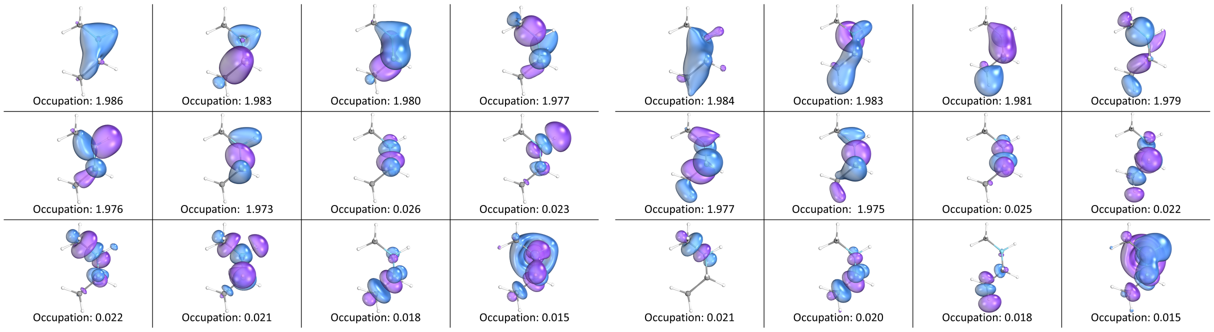

Figure S13: APC(12,12) selected active orbitals for the product in reaction MR_3361_1 with the cc-pVDZ (left) and cc-pVTZ (right) basis sets.

2.8 Case Studies: MR_619998_2

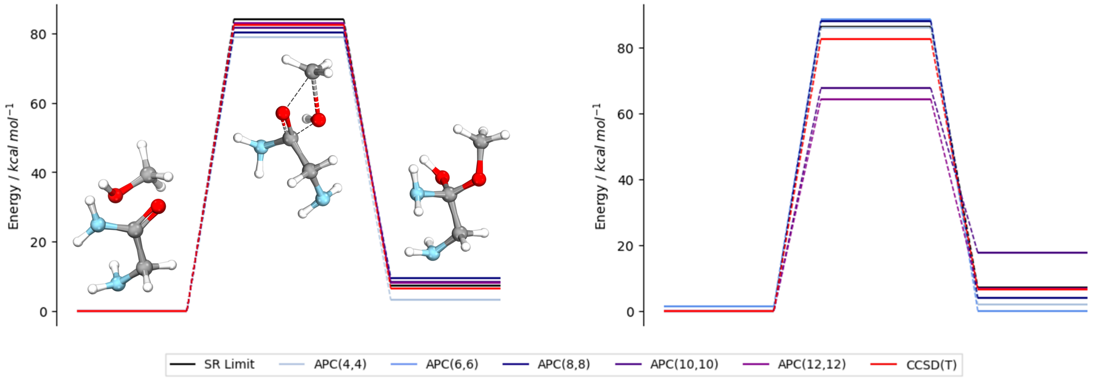

Figure S14: Reaction MR_619998_2 (hemiacetal formation from methanol and glycinamide) with the cc-pVDZ basis set. Active space dependence of tPBE (left) and NEVPT2 (right) shown with HF-PBE/MP2, APC(4,4), APC(6,6), APC(8,8), APC(10,10), and APC(12,12) results with each method. CCSD(T) is included as a reliable reference for single reference reactions. Energies are calculated relative to the lowest energy state.

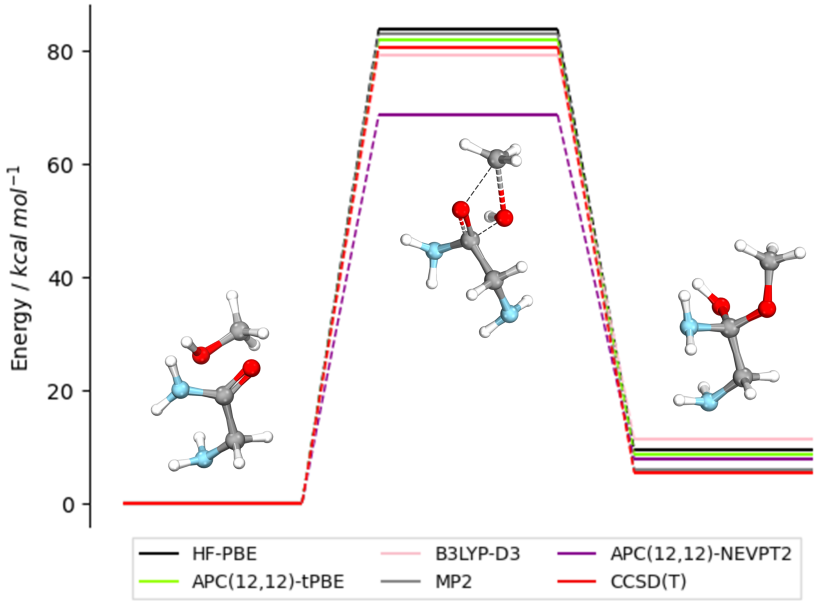

Figure S15: Reaction MR_619998_2 (hemiacetal formation from methanol and glycinamide) with the larger cc-pVTZ basis. APC(12,12)-tPBE (green), APC(12,12)-NEVPT2 (purple), MP2 (grey), HF-PBE (black), B3LYP-D3 (pink), and CCSD(T) (red) energy diagrams are given.

Table S6: Reaction, forward activation, and reverse activation energies for reaction MR_619998_2 as a function of APC active space size with the cc-pVDZ basis. The NEVPT2 result is shown to converge to the single-reference limit as the active space size is decreased.

Active Space	Method	ΔE	E_{af}	E_{ar}
SR	HF-PBE	7.3	84.2	76.9
	MP2	7.1	86.2	79.1
	B3LYP-D3	9.4	79.0	69.6
	CCSD(T)	6.4	82.6	76.2
APC(12,12)	tPBE	8.0	83.2	75.2
	NEVPT2	6.8	64.3	57.5
APC(10,10)	tPBE	8.3	81.8	73.5
	NEVPT2	17.6	67.6	50.0
APC(8,8)	tPBE	9.5	80.3	70.8
	NEVPT2	3.9	88.0	84.1
APC(6,6)	tPBE	5.7	80.4	74.7
	NEVPT2	-1.4	87.1	88.5
APC(4,4)	tPBE	3.1	79.0	75.9
	NEVPT2	1.9	85.6	83.7

Table S7: Reaction and forward activation energies for reaction MR_619998_2 with the larger cc-pVTZ basis set.

Active Space	Method	ΔE	E_{af}	E_{ar}
SR	HF-PBE	9.3	83.8	74.5
	MP2	5.9	82.9	77.0
	B3LYP-D3	11.3	79.2	67.9
	CCSD(T)	5.3	80.6	75.3
APC(12,12)	tPBE	8.5	82.0	73.5
	NEVPT2	7.6	68.6	61.0

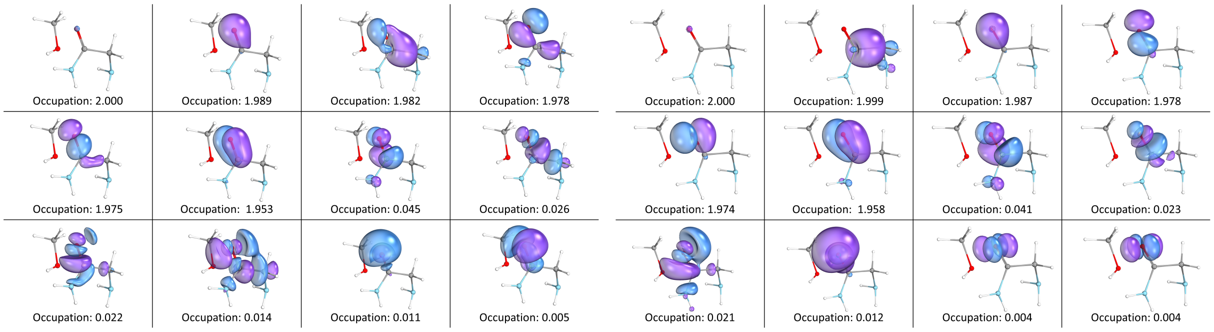

Figure S16: APC(12,12) selected active orbitals for the reactant in reaction MR_619998_2 with the cc-pVDZ (left) and cc-pVTZ (right) basis sets.

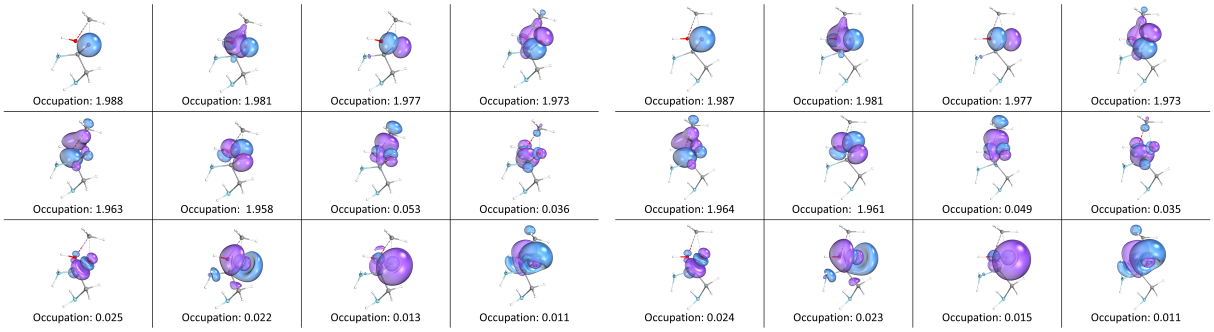

Figure S17: APC(12,12) selected active orbitals for the transition state in reaction MR_619998_2 with the cc-pVDZ (left) and cc-pVTZ (right) basis sets.

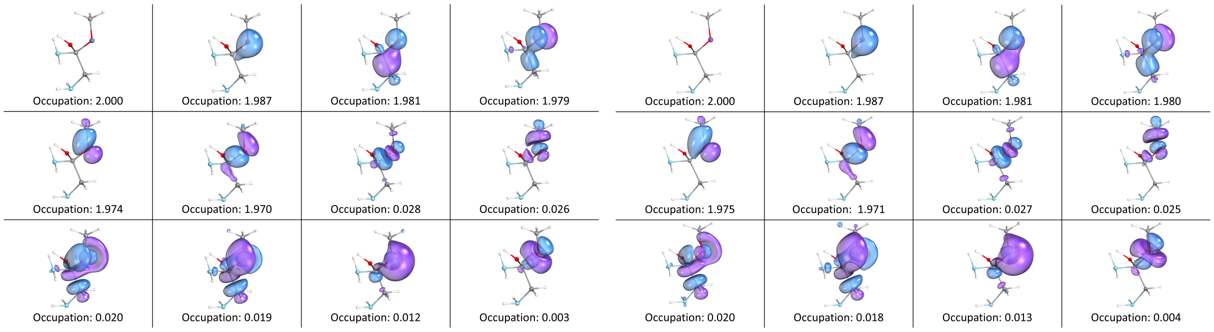

Figure S18: APC(12,12) selected active orbitals for the product in reaction MR_619998_2 with the cc-pVDZ (left) and cc-pVTZ (right) basis sets.

2.9 Case Studies: MR_186317_0

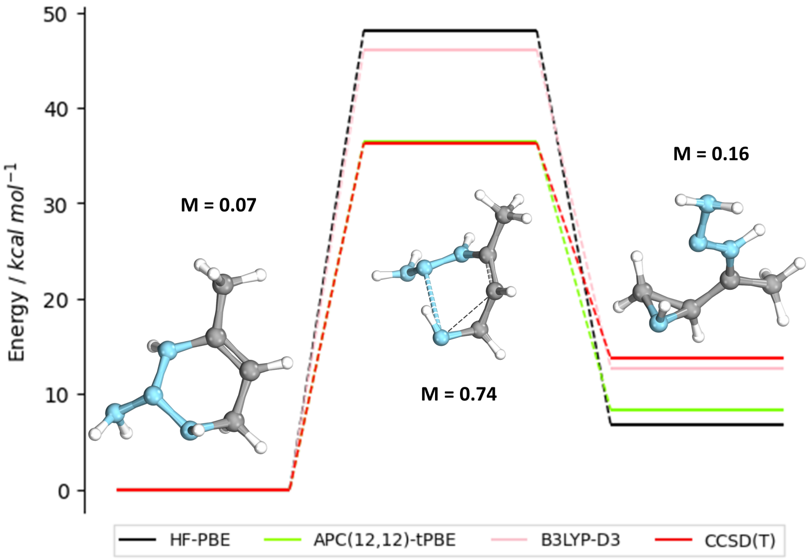

Figure S19: Reaction MR_186317_0 (ring-opening/ring-closing reaction of $N_4C_4H_{10}$) with the larger cc-pVTZ basis. APC(12,12)-tPBE (green), HF-PBE (black), B3LYP-D3 (pink), and CCSD(T) (red) energy diagrams are given.

Table S8: Reaction, forward, and reverse activation energies for reaction MR_186317_0 with all methods used with the cc-pVDZ basis. Here SR are single reference methods and APC(12,12) refers to results using APC selected active spaces of size (12,12).

Active Space	Method	ΔE	E_{af}	E_{ar}
SR	HF-PBE	7.5	48.0	40.5
	MP2	11.5	55.0	43.5
	B3LYP-D3	13.5	48.6	35.1
	CCSD(T)	13.6	33.0	19.4
APC(12,12)	tPBE	8.9	36.2	27.3
	NEVPT2	14.1	40.4	26.3

Table S9: Reaction, forward, and reverse activation energies for reaction MR_186317_0 with the larger cc-pVTZ basis set.

Active Space	Method	ΔE	E_{af}	E_{ar}
SR	HF-PBE	6.7	48.1	41.4
	MP2	11.2	56.4	45.2
	B3LYP-D3	12.7	46.1	33.4
	CCSD(T)	13.7	36.3	22.6
APC(12,12)	tPBE	8.3	36.4	28.1
	NEVPT2	5.4	31.0	25.6

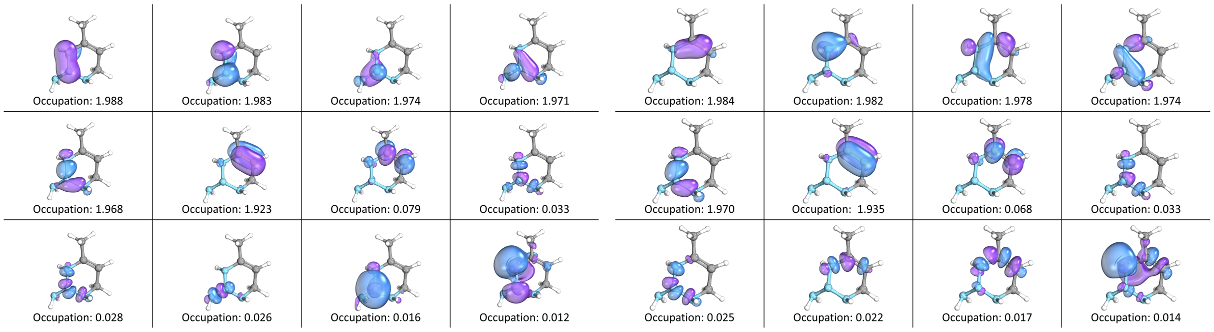

Figure S20: APC(12,12) selected active orbitals for the reactant in reaction MR_186317_0 with the cc-pVDZ (left) and cc-pVTZ (right) basis sets.

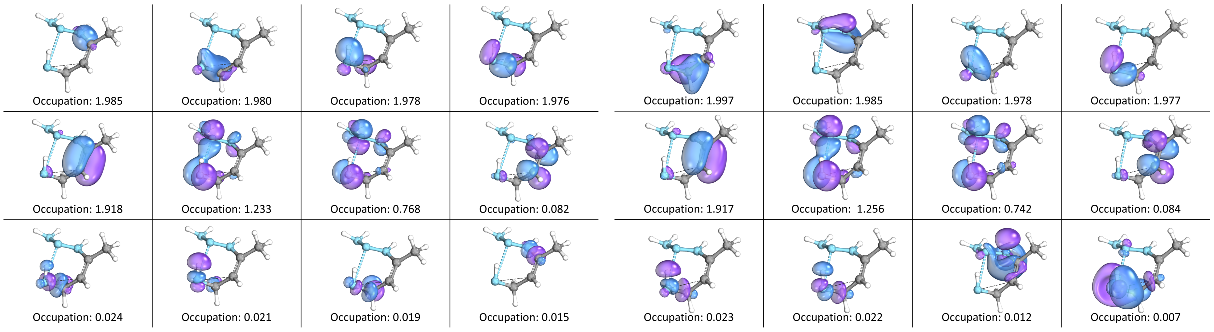

Figure S21: APC(12,12) selected active orbitals for the transition state in reaction MR_186317_0 with the cc-pVDZ (left) and cc-pVTZ (right) basis sets.

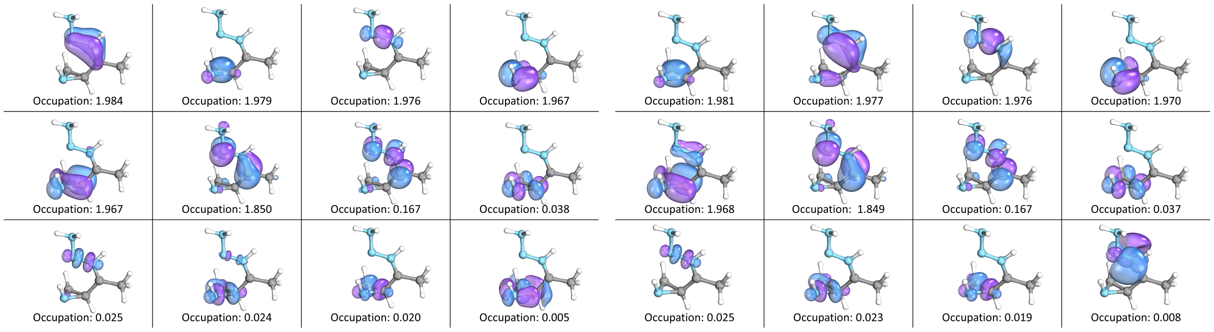

Figure S22: APC(12,12) selected active orbitals for the product in reaction MR_186317_0 with the cc-pVDZ (left) and cc-pVTZ (right) basis sets.

2.10 Case Studies: MR_673407_0

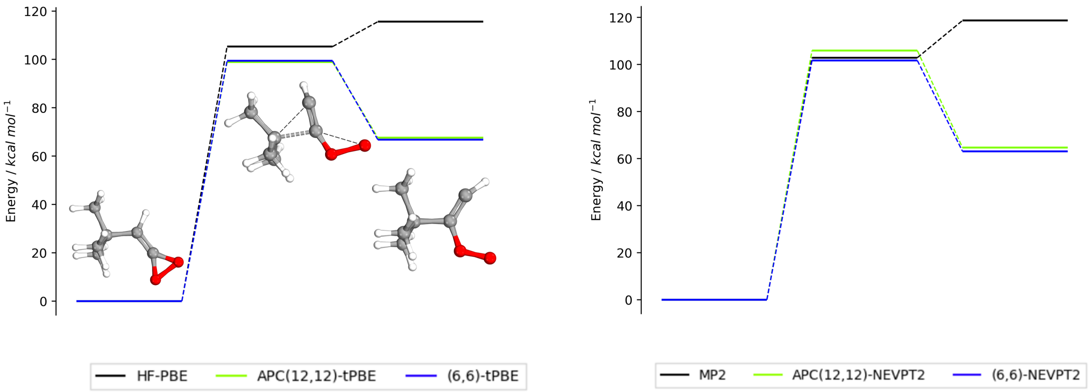

Figure S23: Reaction MR_673407_0 with the cc-pVDZ basis set. Active space dependence of tPBE (left) and NEVPT2 (right) shown with HF-PBE/MP2, APC(12,12) and (6,6) active spaces, where the latter is selected from the most correlated orbitals in the APC(12,12) active space.

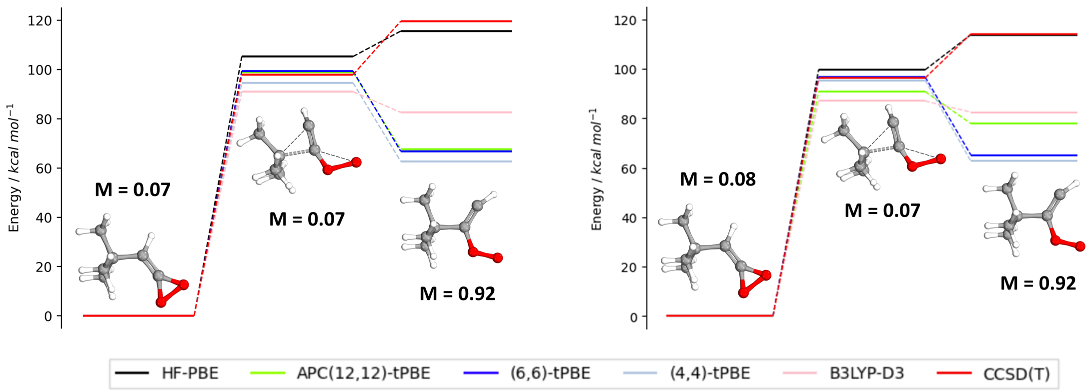

Figure S24: Reaction MR_673407_0 (ring opening of 3-membered heterocycle) with the cc-pVDZ (left) and cc-pVTZ (right) basis sets. APC(12,12)-tPBE (green), (6,6)-tPBE (blue), (4,4)-tPBE (light blue), HF-PBE (black), B3LYP-D3 (pink) and CCSD(T) (red) energy diagrams are given. (4,4) and (6,6) are energetics resulting from selecting the 4 or 6 most correlated orbitals from the APC(12,12) active space and carrying out a CASCI calculation. In the larger basis, increased ASIE is found, which can be greatly reduced by recalculating energetics with only the most correlated orbitals in the active space. This is seen in the improved agreement between (4,4)-tPBE results across basis sets when compared to APC(12,12)-tPBE results, which deviate significantly between cc-pVDZ and cc-pVTZ.

Table S10: Reaction, forward, and reverse activation energies for reaction MR_673407_0 with all methods used in the cc-pVDZ basis. Here SR are single reference methods and APC(12,12) refers to results using APC selected active spaces of size (12,12). (4,4) and (6,6) are energetics resulting from selecting the 4 or 6 most correlated orbitals from the APC(12,12) active space and carrying out a CASCI calculation.

Active Space	Method	ΔE	E_{af}	E_{ar}
SR	HF-PBE	115.6	105.3	-10.3
	MP2	118.7	102.9	-15.8
	B3LYP-D3	82.6	91.0	8.5
	CCSD(T)	119.3	97.9	-21.4
(4,4)	tPBE	62.7	94.6	31.9
	NEVPT2	60.1	96.5	36.4
(6,6)	tPBE	66.8	99.4	32.6
	NEVPT2	63.1	101.7	38.6
APC(12,12)	tPBE	67.6	98.9	31.3
	NEVPT2	64.7	105.9	41.2

Table S11: Reaction, forward, and reverse activation energies for reaction MR_673407_0 with the larger cc-pVTZ basis set. (4,4) and (6,6) are energetics resulting from selecting the 4 or 6 most correlated orbitals from the APC(12,12) active space and carrying out a CASCI calculation. The (4,4)-tPBE results with the cc-pVTZ basis align well with the (4,4) results with the cc-pVDZ basis. This supports the conclusion that the deviation between the two basis with the APC(12,12) active spaces is largely due to different lowly correlated orbitals being selected to the active space with each basis set.

Active Space	Method	ΔE	E_{af}	E_{ar}
SR	HF-PBE	113.8	99.6	-14.2
	MP2	117.8	101.3	-16.5
	B3LYP-D3	82.5	87.0	4.5
	CCSD(T)	114.1	96.3	-17.8
(4,4)	tPBE	63.7	95.4	31.7
	NEVPT2	61.9	86.6	24.7
(6,6)	tPBE	65.2	96.7	31.2
	NEVPT2	62.0	88.4	26.4
APC(12,12)	tPBE	77.9	90.8	12.9
	NEVPT2	68.0	90.3	22.3

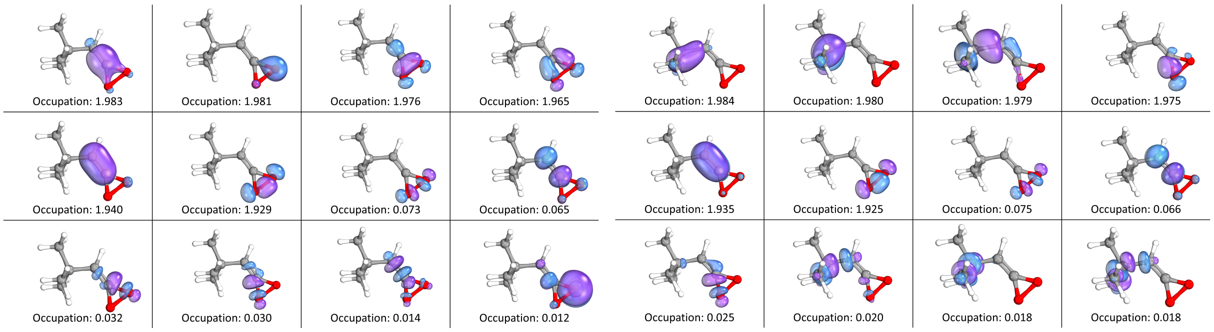

Figure S25: APC(12,12) selected active orbitals for the reactant in reaction MR_673407_0 with the cc-pVDZ (left) and cc-pVTZ (right) basis sets.

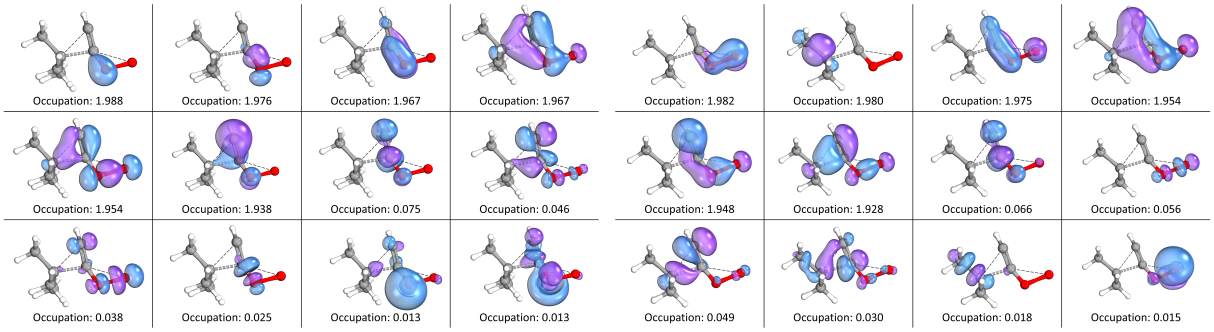

Figure S26: APC(12,12) selected active orbitals for the transition state in reaction MR_673407_0 with the cc-pVDZ (left) and cc-pVTZ (right) basis sets.

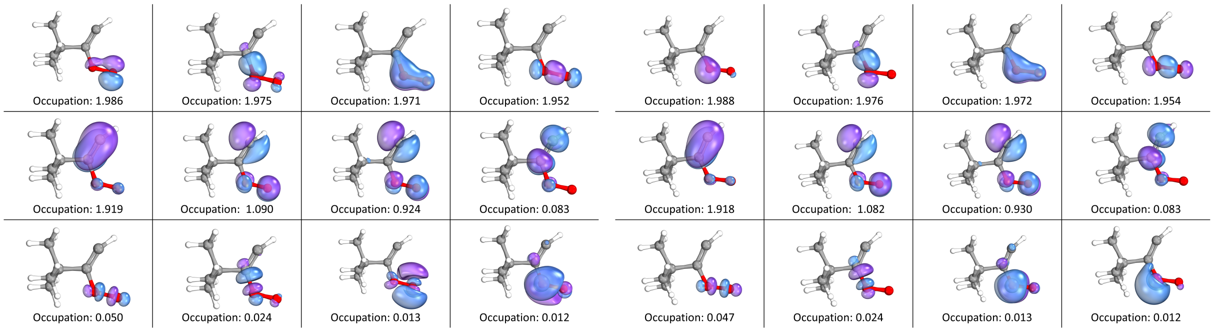

Figure S27: APC(12,12) selected active orbitals for the product in reaction MR_673407_0 with the cc-pVDZ (left) and cc-pVTZ (right) basis sets. The biradical nature of the product is well-described.

Table S12: Reaction, forward, and reverse activation energies for reaction MR_673407_0 with the cc-pVTZ basis set as a function of N, the number of virtual orbital removal steps in the APC scheme.

N	Method	ΔE	E_{af}	E_{ar}
0	tPBE	77.9	90.8	12.9
	NEVPT2	68.0	90.3	22.3
2	tPBE	77.9	90.8	12.9
	NEVPT2	68.0	90.3	22.3
4	tPBE	77.9	90.8	12.9
	NEVPT2	68.0	90.3	22.3
6	tPBE	79.2	92.1	12.9
	NEVPT2	71.9	94.2	22.3
8	tPBE	79.2	92.1	12.9
	NEVPT2	71.9	94.2	22.3
10	tPBE	77.9	90.8	12.9
	NEVPT2	68.0	90.3	22.3

References

(S1) Mitchell, E. C.; Scott, T. R.; Bao, J. J.; Truhlar, D. G. Application of Multiconfiguration Pair-Density Functional Theory to the Diels–Alder Reaction. *J. Phys. Chem. A* **2022**, *126*, 8834–8843.

(S2) Lischka, H.; Ventura, E.; Dallos, M. The Diels–Alder Reaction of Ethene and 1,3-Butadiene: An Extended Multireference Ab Initio Investigation. *ChemPhysChem* **2004**, *5*, 1365–1371.

(S3) Pandharkar, R.; Hermes, M. R.; Truhlar, D. G.; Gagliardi, L. A New Mixing of Nonlocal Exchange and Nonlocal Correlation With Multiconfiguration Pair-Density Functional Theory. *J. Phys. Chem. Lett.* **2020**, *11*, 10158–10163.

(S4) King, D. S.; Hermes, M. R.; Truhlar, D. G.; Gagliardi, L. Large-Scale Benchmarking of Multireference Vertical-Excitation Calculations via Automated Active-Space Selection. *J. Chem. Theory Comput.* **2022**, *18*, 6065–6076.
