# Supplementary material for: Organic Reactivity Made Easy and Accurate with Automated Multireference Calculations
Source: ACS Cent Sci. 2024 Mar 27;10(4):833–41. doi: 10.1021/acscentsci.3c01559 (PMC11046455; doi:10.1021/acscentsci.3c01559)
Supplement: Supplementary file 2 — oc3c01559_si_002.pdf [file oc3c01559_si_002.pdf]

oc-2023-01559s.R1

Name: Peer Review Information for "Organic Reactivity Made Easy and Accurate with Automated Multireference Calculations"

First Round of Reviewer Comments

Reviewer: 1

Comments to the Author

The manuscript addresses an issue which for a long time has been holding back the general usability of multi-configurational wave function models -- the selection of the active space. The remedy is the approximate pair coefficient selection (APC), in this paper this approach is put to task for a large set of randomly selected reactions for medium sized molecular systems. The selection of the active space over a reaction path should be consistent, this would require that the active space is investigated for several molecular structures and a subsequent union of the proposed active spaces are selected -- this is avoided in this paper. The authors accept the inconsistency -- resulting in a active space inconsistency error (ASIE) -- but argue the such an error is smaller for the MC-PDFT approach as compared to CASSCF/CASPT2,NEVPT2 approach.The authors test this hypothesis for some 908 randomly selected reactions in association with a cc-PVDZ basis set and compared against B3LYP-D3 and CCSD(T) results -- both being single reference methods. The various molecular structure are furthermore ranked into three groups depending on how multi-configurational the associated CASSCF wve function is according to the APC scheme. Here the extreme ranks are cases of insignificant or massive multi-configurational nature. Here, for example, one expect that the APC-tPBE approach should agree with with the single reference methods in the case of wave functions with minimal multi-configurational nature. Additionally, as the wavefunctions get more and more multiconfigurational the deviation to the single reference models increase -- just as expected. The authors provide convincing data to prove their point and additionally bring up five explicit cases in which the results are discussed and analysed in details. This is all very interesting and will only require some additional corrections -- some being trivial, some being a bit more elaborate. Lets start with the trivial one,

- the acronym APC is used before it is explained

- the strange behavior that the NEVPT2 with the smaller active space is more correct than the once with the larger has to be understood. As the active space is increased the CASSCF step should ultimately converge to the full CI result, making the NEVPT2 step redundant. What is the problem in the cases mentioned in the paper? Is the (4,4) active space selection just an accidental selection with gives a better result? What happens with a (2,2), (6,6) and (8,8) selection?

- there has to be some data presented that supports that the observations also are valid at the cc-pVTZ level. Here it is also important to note that the B3LYP-D3 and the CCSD(T) references converge with respect to the basis sets in a different way. How does a larger basis set affect the APC procedure -- the number of virtual orbitals increase and are harder for identify as candidates for the active space.

- What happens with the APC and the results as N -- iterations in which the virtual orbitals are remove -- increase?

- Finally, it would be nice if there was some comparison with experimental results -- at the end this is what the authors hope to reproduce.

After the modifications of the draft in according with the suggestions above I recommend publication.

Reviewer: 2

Comments to the Author

In this manuscript, the authors present a high-throughput study of the reaction and activation energies of 908 simple chemical reactions of HCNO compounds with the multi-reference APC(12,12)-PDFT method. DFT, CCSD(T) and NEVPT2 results are provided for comparison. The aim is to have an automatic black-box approach for all types of reactions. The authors argue that 68% of the reactions are significantly multireference. They also show that APC(12,12)-PDFT is appreciably less sensitive to the active space inconsistency error than NEVPT2/CASSCF(12,12) for single-reference reactions. The study is quite strange. It mainly shows that this type of high-throughput calculations is possible, but the results are not much used (only in a single figure; all the other results are examples) and the big problem is that there are no clear reference calculations, so the reliability of the results is unknown. In fact, the basis sets are so small (cc-pVDZ) that it is quite certain that none of the results are reliable. The main problem discussed is whether the too-large active space used for all reaction deteriorates the results or not. Still, I think the manuscript may be published, although a more computational journal (JCTC) would probably be more appropriate.

1. What is the “on-top” density?
2. APC should be explained the first time used.
3. Why is the D3 and not D4 dispersion correction used? What is the damping with D3?
4. How is the user-defined size requirement decided?
5. (12,12) and (A,B) should be explained first time used.

6. I cannot see how the procedure described at the turn of p. 6–7 lead to a consistent active space between geometries. Different geometries are not even mentioned. It only gives the user-defined number of orbitals.

7. How are rotation of orbitals out of the active space handled? This is most likely still a problem.

8. “break two form one” and “break two form two” need to be explained.

9. Why was such a small basis set (cc-VDZ) used for the energy calculations (smaller than for geometries)? The authors argue that the calculations are as fast as DFT, for which there is not problem to obtain TZ energies. None of the methods used will give reliable energies at the DZ level.

10. What is MR-AQCC? Is it accurate enough to give reference energies? The study is 20 years old.

11. Do all methods in Fig 1 employ the same basis set?

12. CCSD(T) and pure PBE-D3 (or B3LYP-D3) results should be added to Fig 1.

13. What does “for a total of 68% overall” mean?

14. APC(12,12)-PDFT results should be added to Fig 1.

15. As far as I can see, the APC(12,12)-tPBE result in Fig 3 differs from the CCSD(T) reference by ~4.5 kcal, considerably worse than B3LYP of APC(4,4)-tPBE, i.e. APC(12,12)-tPBE still shows a significant ASIE.

16. I do not understand the text “and in much better agreement with the CCSD(T) reference values for the single-reference reactant and product”. Did not the authors just argue that the CCSD(T) results of the product are unreliable (as also shown in the figure)?

17. Is it not possible to automatically also select the size of the active space? A (12,12) space seems completely arbitrary and a wase of computational resources.

18. Where does the suggestion “which successfully reproduces the single-reference limit with ASIE of ±2.2 kcal/mol” come from?

19. Are the B3LYP-D3 results obtained with the DZ or TZ basis set?

Reviewer: 3

Comments to the Author

I enjoyed reading this paper. The effort to provide black box MR methods for organic reactivity is commendable and forward thinking. The paper is also well-written, well-structured and of the right length. I believe that it will create a fair bit of interest in the community.

I do not intend to nitpick on every single detail of the study. There is, however, one aspect that I feel uncomfortable with: the reference data is created by CCSD(T)/cc-pVDZ(page 8). It is well-known, that CC and DFT converge to the basis set limit at very different rates. Since cc-pVDZ is so very far from the basis set limit, I would argue that its use to generate reference data is nearly meaningless. The deviations from the basis set limit will be pretty much as large or larger than the deviations between different methods. CCSD(T)-F12/cc-pVDZ-F12 would do a much better job or any of the available local CC methods properly CBS extrapolated.

While redoing all of the reference data may not be practical, I think the authors should take at least a subset of small systems and generate proper CBS reference data that they then can compare their APC results to. Readers should know how these deviations compare to the ones one is getting using the more approximate reference data. One system would not be enough to convince me.

But apart from that - very interesting study that I definitely support.

Author's Response to Peer Review Comments:

Journal: ACS Central Science
Manuscript ID: oc-2023-01559s
Title: "Organic Reactivity Made Easy and Accurate with Automated Multireference Calculations"
Authors: Wardzala, Jacob; King, Daniel; Ogunfowora, Lawal; Savoie, Brett; Gagliardi, Laura

Throughout these responses, black text will indicate reviewer's comments, blue our responses, and red changes to the manuscript resulting from those comments.

Reviewer 1

R1-0) Recommendation: Publish in ACS Central Science after minor revisions noted.

Comments:
The manuscript addresses an issue which for a long time has been holding back the general usability of multi-configurational wave function models -- the selection of the active space. The remedy is the approximate pair coefficient selection (APC), in this paper this approach is put to task for a large set of randomly selected reactions for medium sized molecular systems. The selection of the active space over a reaction path should be consistent, this would require that the active space is investigated for several molecular structures and a subsequent union of the proposed active spaces are selected -- this is avoided in this paper. The authors accept the inconsistency -- resulting in a active space inconsistency error (ASIE) -- but argue the such an error is smaller for the MC-PDFT approach as compared to CASSCF/CASPT2,NEVPT2 approach.The authors test this hypothesis for some 908 randomly selected reactions in association with a cc-PVDZ basis set and compared against B3LYP-D3 and CCSD(T) results -- both being single reference methods. The various molecular structure are furthermore ranked into three groups depending on how multi-configurational the associated CASSCF wve function is according to the APC scheme. Here the extreme ranks are cases of insignificant or massive multi-configurational nature. Here, for example, one expect that the APC-tPBE approach should agree with with the single reference methods in the case of wave functions with minimal multi-configurational nature. Additionally, as the wavefunctions get more and more multiconfigurational the deviation to the single reference models increase -- just as expected. The authors provide convincing data to prove their point and additionally bring up five explicit cases in which the results are discussed and analysed in details. This is all very interesting and will only require some additional corrections -- some being trivial, some being a bit more elaborate.

We thank the reviewer for their generally positive view of our work and have addressed their comments below.

R1-1) Let's start with the trivial one, the acronym APC is used before it is explained

We agree this should be corrected and adjusted the text as follows (changes in red):

"We find that combining the approximate pair coefficient active space selection scheme (APC) with MC-PDFT (referred to as APC-PDFT) generates robust results, with APC-PDFT reproducing DFT results for a set of single reference reactions." (Page 5)

R1-2) the strange behavior that the NEVPT2 with the smaller active space is more correct than the once with the larger has to be understood. As the active space is increased the CASSCF step should ultimately converge to the full CI result, making the NEVPT2 step redundant. What is the problem in the cases mentioned in the paper? Is the (4,4) active space selection just an accidental selection with gives a better result? What happens with a (2,2), (6,6) and (8,8) selection?

The reviewer is correct that as the in the FCI limit the NEVPT2 result should converge appropriately. However, the systems shown in figure 3 have 25 and 38 valence orbitals, respectively, placing them far out of reach of the (12,12) spaces treated multiconfigurationally. The worse results with the larger spaces are due to the increased ASIE yielded by NEVPT2 due to inconsistencies in the 2-RDM. We have elaborated on this in a new section in the supporting information:

"Discussion on the Robustness of MC-PDFT Towards ASIE

As shown in several examples throughout the manuscript and supporting information, MC-PDFT appears to have a significantly increased robustness towards ASIE as compared to CASSCF and NEVPT2. The theoretical reasoning for why this is the case is worth discussing in greater detail here. As presented in the introduction, we hypothesize that the greater ASIE of CASSCF and NEVPT2 is due to the unequal energetic contributions of inconsistent orbitals to the 2-RDM between geometries. As MC-PDFT excludes the 2-RDM from its energy expression (except indirectly through the on-top density Π), it is significantly more robust to these orbital inconsistencies.

Additionally, NEVPT2 can often be more prone to ASIE than CASSCF (as shown for MR_3361_1 below) as the perturber states also change as a function of the active orbitals. While it is true that NEVPT2 should converge appropriately in the FCI limit, making the perturbative treatment redundant, the CAS active space size is very far from this limit even for the small-to-medium-sized molecules studied here. For example, the molecules in Figure 3 contain a total of 25 and 38 valence orbitals, respectively, significantly larger than the (12,12) space treated multiconfigurationally. As such, larger APC active spaces can often perform much worse with these active spaces due to ASIE. If larger active spaces are imbalanced or not converged, significant changes in NEVPT2 results relative to smaller, balanced active spaces can occur, highlighting the strong dependence of NEVPT2 on the active space. Comparatively, MC-PDFT is more robust to the active space choice, making it an advantageous method for multireference reactivity calculations.

Below, we provide studies of the ASIE in CASSCF, NEVPT2, and tPBE with increasing active space size for the reactions shown in Figure 3, as well as orbitals and cc-pVTZ results for all case studies shown in the manuscript." (Page S11-S12)

A study of the behavior of NEVPT2 with active spaces between (4,4) and (12,12) is available in the supporting information, and is noted on page 13:

“An in-depth evaluation of the active space dependence of tPBE and NEVPT2 for these two reactions, as well as CASSCF is included in the Supporting Information,” (Page 13)

Figures S10 and S14 give comparison between APC(4,4), APC(6,6), APC(8,8), APC(10,10), and APC(12,12) for tPBE and NEVPT2. They show APC(4,4)-APC(8,8) are in reasonable agreement with the single reference limit, but the additions made to the two larger active spaces result in large deviations from the single reference limit for the NEVPT2 results for the reactions shown in Figure 3.

R1-3) there has to be some data presented that supports that the observations also are valid at the cc-pVTZ level. Here it is also important to note that the B3LYP-D3 and the CCSD(T) references converge with respect to the basis sets in a different way. How does a larger basis set affect the APC procedure -- the number of virtual orbitals increase and are harder for identify as candidates for the active space.

The other reviewers have noted this issue, and we agree it needs to be addressed. We have recomputed the results shown in Figures 3-5 using the cc-pVTZ basis. Overall, we find APC-tPBE to be remarkably consistent in all of the case studies, with visually near-identical correlating orbitals being chosen in all cases. Nearly all energetic results with cc-pVTZ show good qualitative agreement with the trends and comparisons discussed for the cc-pVDZ results found in the main text. However, a large discrepancy is found in the cc-pVTZ description of MR_673407_0, in which the APC(12,12)-tPBE reaction energy changes from 31.3 kcal/mol in the cc-pVDZ basis to 12.9 kcal/mol in the cc-pVTZ basis. We find that this is due to an abnormally large ASIE in the cc-pVTZ basis, which can be eliminated by performing a CASCI calculation in only the (4,4) space of strongly correlating orbitals, which are identical between cc-pVDZ and cc-pVTZ. We believe this approach of recomputing reaction energies using CASCI in only the space of correlating orbitals is promising for further reducing ASIE in APC-tPBE and will be explored in future work. We have added these cc-pVTZ results with visualizations of the active orbitals to the SI and the following text to the manuscript referring the reader to these results:

Results added to Supporting Information:
Figure 3: Figures S11 and S15, Tables S5 and S7
Figure 4: Figure S19, Table S9
Figure 5: Figure S24, Table S11

“To explore basis set dependence, we have investigated the behavior of B3LYP, APC-tPBE, and CCSD(T) in the larger cc-pVTZ basis for the case studies presented in Figures 3-5 (Supporting Information). Overall, we find the APC-tPBE to be remarkably consistent with respect to basis set size, with nearly all results in the cc-pVDZ basis set being well-reproduced in the larger cc-pVTZ basis qualitatively similar correlating orbitals being chosen in all cases. However, a large discrepancy is found in the cc-pVTZ description of MR_673407_0, in which the APC(12,12)-tPBE reaction energy of the diradical reaction energy changes from 31.3 kcal/mol in the cc-pVDZ basis to 12.9 kcal/mol in the cc-pVTZ basis. We find that this discrepancy is due to an abnormally large ASIE in the cc-pVTZ basis, which can be eliminated by executing a CASCI in

only the (4,4) active space of correlating orbitals (visually identical to those of the cc-pVDZ basis), which largely reproduces the results shown in Figure 5. This process of recomputing reaction energies using CASCI calculations in only the space of correlating orbitals is promising for further reducing ASIE in APC-tPBE and will be explored in future work." (Page 15-16)

R1-4) What happens with the APC and the results as N -- iterations in which the virtual orbitals are remove -- increase?

The N parameter was introduced in previous papers to balance the selection of doubly occupied and virtual orbitals. Here, because we choose exactly 6 virtual orbitals and 6 doubly occupied orbitals for each system, we expect that the N parameter has a minimal effect. We have tested this by performing calculations with varied N, from 0 to 10, in the cc-pVTZ basis for the system in figure 5 Figure 5, which has a minimal effect. We have now included these results in the Supporting Information and noted this in the Methods section:

Table S12 added to Supporting Information

"However, this parameter has less impact due to the fixed active space size we employ here to enforce active space size consistency between different geometries (described below)." (Page 7)

R1-5) Finally, it would be nice if there was some comparison with experimental results -- at the end this is what the authors hope to reproduce.

We agree that comparison with experiment would bolster our results. However, this comparison is generally impossible given the computationally enumerated nature of the RGD-1 dataset. In other words, experimental data for these reactions do not exist. As such, we have done our best to compute reference values using CCSD(T) when the geometries are sufficiently single reference (Figures 3 and 4) and compare to different multiconfigurational results when this is not the case (Figures 1 and 5).

However, we argue that Figure 1 should suffice as a genuine experimental comparison, as the benchmark MR-AQCC results found by Lischka *et al.* found the MR-AQCC results to be in good agreement with the experimentally accepted reaction pathway. We have now noted this on Page 9:

"The study from Lischka *et al.* showed the MR-AQCC results to be in good agreement with experiment for the accepted reaction pathway." (Page 9)

Reviewer 2

R2-0) Recommendation: Publish elsewhere JCTC

Comments:
In this manuscript, the authors present a high-throughput study of the reaction and activation energies of 908 simple chemical reactions of HCNO compounds with the multi-reference

APC(12,12)-PDFT method. DFT, CCSD(T) and NEVPT2 results are provided for comparison. The aim is to have an automatic black-box approach for all types of reactions. The authors argue that 68% of the reactions are significantly multireference. They also show that APC(12,12)-PDFT is appreciably less sensitive to the active space inconsistency error than NEVPT2/CASSCF(12,12) for single-reference reactions. The study is quite strange. It mainly shows that this type of high-throughput calculations is possible, but the results are not much used (only in a single figure; all the other results are examples) and the big problem is that there are no clear reference calculations, so the reliability of the results is unknown. In fact, the basis sets are so small (cc-pVDZ) that it is quite certain that none of the results are reliable. The main problem discussed is whether the too-large active space used for all reaction deteriorates the results or not. Still, I think the manuscript may be published, although a more computational journal (JCTC) would probably be more appropriate.

While we take the point that the paper largely shows that these types of high-throughput calculations are possible, this achievement represents a significant milestone in the development of multiconfigurational approaches. It has long been known that multiconfigurational methods are important for getting good results in many organic transition states, and demonstrating the robustness and reliability of these approaches opens the door to more accurate descriptions of chemical systems at a large scale.

Furthermore, while we agree that more can always be done, we have done our best to show that our methods compare favorably to benchmark calculations throughout the paper. Figure 1 shows the striking agreement of our automated procedure with benchmark Diels-Alder results, which are in agreement with the accepted experimental reaction energetics. Figure 3 demonstrates that the automated approach compares favorably to benchmark CCSD(T) results in single-reference systems, while Figures 4 and 5 show the important deviations of the automated multiconfigurational method with DFT and CCSD(T), respectively in systems with multiconfigurational character.

As such, we believe that our paper presents an important contribution to the quantum chemical community in the broadest sense and is thus suitable for publication in ACS Central Science. Moreover, this paper also has a pedagogical purpose. It teaches to those who perform quantum chemical calculations that one should always try to understand the meaning of a computational result and not only generate numbers. The hope is that these types of calculations become more user friendly and more people start performing them. We have done our best to respond to the reviewer’s comments below.

R2-1) What is the “on-top” density?

The on-top pair density, Π, describes the likelihood of finding two electrons at a given point in space and is derived from the two-particle density matrix. As noted in equation 5, it is used in the MC-PDFT energy expression in addition to the standard electron density. We have added the following line to the text where the term is introduced:

“The on-top pair density, derived from the two-particle density matrix, describes the probability of finding two electrons at the same point in space.” (Page 5)

R2-2) APC should be explained the first time used.

This has been addressed in response to R1-1:

"We find that combining the approximate pair coefficient active space selection scheme (APC) with MC-PDFT (referred to as APC-PDFT) generates robust results, with APC-PDFT reproducing DFT results for a set of single reference reactions." (Page 5)

R2-3) Why is the D3 and not D4 dispersion correction used? What is the damping with D3?

B3LYP with D3 dispersion corrections were used in the original data (RGD1) and as a result was used again here. The D3 dispersion was used as implemented in PySCF through the dftd3 package (https://github.com/dftd3/simple-dftd3) with zero damping.

R2-4) How is the user-defined size requirement decided?

The size of (12,12) active spaces was chosen based off knowledge of the size of the reactions being studied. This is addressed in the manuscript:

"To account for the cases with the most multiconfigurational character, we have chosen large APC(12,12) active spaces for each state in these reactions. This active space size is significantly larger than necessary for most reactions in the dataset, resulting in inconsistent but unimportant orbitals between the reactants and products of some reactions." (Page 10)

R2-5) (12,12) and (A,B) should be explained first time used.

We have updated the text to include the following explanation:

"Candidate HF orbitals are then ranked in importance by their orbital entropies, with this ranking used to choose an active space meeting some user-defined size requirement (e.g., a 12 electron in 12 orbital or (12,12) active space). Here, to select consistent active space sizes between geometries we employ a simple size requirement in which for an (A,B) active space, where A and B are the number of active electrons and orbitals respectively…" (Page 7)

R2-6) I cannot see how the procedure described at the turn of p. 6–7 lead to a consistent active space between geometries. Different geometries are not even mentioned. It only gives the user-defined number of orbitals.

Indeed, the only fixed variables between different geometries is the number of electrons and orbitals in the active space. The reason that the APC scheme helps to choose fairly consistent active spaces between geometries is because it is a function of the Fock and exchange matrix elements, which change adiabatically as a function of the geometry; we have now noted this reasoning on page 7:

“As the pair coefficients are generated from Fock and exchange matrix elements which change adiabatically with the molecular geometry, the APC scheme aims to select moderately consistent (but not exactly consistent) active spaces across the reaction coordinate.” (Page 7)

However, as noted, the procedure will not yield fully consistent active spaces between geometries, and thus introduces active space inconsistency error (ASIE) into the result. However in this work, we show that the MC-PDFT is far more robust to this error than other post-MCSCF methods, making it possible to perform such an automated active space selection and still yield valid results.

R2-7) How are rotation of orbitals out of the active space handled? This is most likely still a problem.

In this work, to enable the high-throughput approach, geometries are not interpolated between each other as is usually done (as noted on page 3). As such, orbitals are not “rotated out of the active space” in the usual sense. Instead, the APC scheme simply selects slightly different sets of orbitals at one geometry and another. These inconsistencies result in ASIE, we show that MC-PDFT is significantly more robust to this error than other post-MCSCF methods (Figures 2 and 3), enabling the high-throughput application of this approach.

R2-8) “break two form one” and “break two form two” need to be explained.

We have updated the text to include the following explanation:

“The B2F1 reactions, which break two bonds and form one bond as the reaction progresses from reactant to product, have an increased likelihood of showing MR character due to the uneven number of bonds formed and broken in the reaction, whereas the B2F2 reactions, which have two bonds broken and two bonds formed throughout the reaction, have closed-shell reactants and products (Supporting Information).” (Page 8)

R2-9) Why was such a small basis set (cc-VDZ) used for the energy calculations (smaller than for geometries)? The authors argue that the calculations are as fast as DFT, for which there is not problem to obtain TZ energies. None of the methods used will give reliable energies at the DZ level.

We have addressed this comment in response to R1-3. Figures 3-5 have all been recalculated at the cc-pVTZ level, which qualitatively reproduce the results found at the cc-pVDZ level (with minor differences as noted in the response to R1-3).

R2-10) What is MR-AQCC? Is it accurate enough to give reference energies? The study is 20 years old.

MR-AQCC stands for multireference averaged quadratic coupled cluster, which we have now clarified:

“multireference averaged quadratic coupled cluster (MR-AQCC) calculations” (Page 9)

This method is shown to give accurate reference energies where experimental results are available in the cited study. Despite the 20-year-old nature of the study, the energetics and transition and intermediate state geometries obtained have been further validated in later work. Also see R1-5 for related comments about MR-AQCC and accuracy relative to experiment for the Diels Alder reaction.

R2-11) Do all methods in Fig 1 employ the same basis set?

All results calculated for this work use the cc-pVDZ basis. The hand selected MC-PDFT results, from Truhlar et al., also use the cc-pVDZ basis. The MR-AQCC results, from Lischka et al., are calculated with the 6-311G** basis.

R2-12) CCSD(T) and pure PBE-D3 (or B3LYP-D3) results should be added to Fig 1.

The Diels-Alder reaction is one of the most well-studied reactions in quantum chemistry, and a detailed review of several approaches can be found here: https://doi.org/10.1021/jp035501w. We emphasize that the cited MR-AQCC results of Lischka et al. are widely regarded as the most accurate, and are in agreement with the experimentally accepted mechanism, as noted by https://doi.org/10.1021/acs.jpca.2c06433.

While we appreciate the reviewer's input, we believe that adding other methods to Figure 1 would take away from the point of this figure which is to simply confirm that our automated scheme reproduces the hand-selected multiconfigurational results of a previous study, which reproduces that of a widely accepted benchmark result. We do not wish to relitigate the accuracy of different methods in modeling this reaction. However, CCSD(T) and B3LYP-D3 results have been added to the Diels-Alder energetics section of the SI.

R2-13) What does "for a total of 68% overall" mean?

This total indicates that 68% of the reactions studied have at least one state (reactant, transition state, or product) that has high multiconfigurational character (M diagnostic > 0.1). This is updated as follows in the text:

"Our calculations show that this set of reactions shows a broad distribution of multiconfigurational character as measured by the M-diagnostic (Supporting Information), with 32% of reaction energies and 63% of activation energies demonstrating significant multiconfigurational character (M > 0.1), for a total of 68% of reactions exhibiting such character in at least one state overall." (Page 10)

R2-14) APC(12,12)-PDFT results should be added to Fig 1.

APC(12,12)-PDFT results are included in the SI (Table S1), and show little deviation from APC(6,6)-PDFT results. This is now referenced in the manuscript on page 10:

“Results with an APC(12,12) active space as well as KS-DFT and CCSD(T) are reported in the Supporting Information. The larger active space results are in good agreement with the APC(6,6) performance.” (Page 10)

R2-15) As far as I can see, the APC(12,12)-tPBE result in Fig 3 differs from the CCSD(T) reference by ~4.5 kcal, considerably worse than B3LYP of APC(4,4)-tPBE, i.e. APC(12,12)-tPBE still shows a significant ASIE.

Yes, there is non-negligible ASIE in this reaction: APC(12,12) predicts a reaction energy of -6.7 kcal/mol, while the single-reference limit of HF-PBE predicts a reaction energy of -4.4 kcal/mol (Table S4). This gives an ASIE of 2.3 kcal/mol, which is more-or-less the average we give for the ASIE estimate of our automated scheme (2.2 kcal/mol, Figure 2). We do not claim that there is no ASIE, but that it is significantly reduced to acceptable levels. Evidently the difference between B3LYP and HF or B3LYP and CCSD(T) can be much greater than this in single reference cases (Figure 3, right). However, we have noted that the ASIE in this case lies somewhat above chemical accuracy:

“As is seen, APC-tPBE successfully reproduces HF-PBE to within 3 kcal/mol, a result that is similarly in-line with B3LYP-D3 and CCSD(T). Though this deviation is slightly larger than chemical accuracy, it presents a substantial improvement over APC(12,12)-NEVPT2, which shows a clear deviation from all other methods, overestimating the energy of the reactant by roughly 30 kcal/mol, despite using the same underlying APC-CASSCF wave functions as APC-tPBE.” (Page 12-13)

R2-16) I do not understand the text “and in much better agreement with the CCSD(T) reference values for the single-reference reactant and product”. Did not the authors just argue that the CCSD(T) results of the product are unreliable (as also shown in the figure)?

We appreciate the reviewer’s close reading. The original statement was indeed erroneous. It should refer to the single-reference reactant and transition state, not the product. It has been updated as follows:

“… and in much better agreement with the CCSD(T) reference values for the single-reference reactant and transition state.” (Page 15)

R2-17) Is it not possible to automatically also select the size of the active space? A (12,12) space seems completely arbitrary and a wase of computational resources.

While one can imagine also selecting the size of the active space, there are good reasons for not doing this as discussed in the original APC paper (https://doi.org/10.1021/acs.jctc.1c00037). In simple terms, methods that attempt to determine the number of orbitals and electrons are often highly basis dependent, and due to the exponential scaling, this can quickly result in a computationally infeasible calculation. Furthermore, this choice also makes APC easier to adapt for different active space methods which have different active space size limitations (e.g. CASSCF vs. DMRG). We have now discussed this on Page 6:

“We note that APC is a ranked-orbital approach, where the user defines a maximum active space size. This method allows the practitioner to prevent the selection scheme from picking active spaces larger than are computationally feasible and it also allows for flexibility towards solvers with different practical size limitations (i.e. CAS vs. DMRG). The drawback is that the user has to define this maximum size manually which can result in an unnecessarily large active space.” (Page 6)

R2-18) Where does the suggestion “which successfully reproduces the single-reference limit with ASIE of ±2.2 kcal/mol” come from?

This number comes from reactions with low multiconfigurational character (M<0.05) shown in Figure 2, which are considered reasonably single reference. For these reaction and activation energies, the average deviation of APC-tPBE from the single-reference limit (HF-PBE) is 2.2 kcal/mol. See the following discussion from the manuscript:

“In the cases with low multiconfigurational character, M < 0.05, tPBE successfully reproduces the single-reference limit with a mean deviation of +/-1.8 kcal/mol for ΔE and +/-2.8 kcal/mol for E_a,with an average between these two of +/-2.2 kcal/mol.” (Page 11)

R2-19) Are the B3LYP-D3 results obtained with the DZ or TZ basis set?

Reported results are with the cc-pVDZ basis. As addressed in previous comments cc-pVTZ results have been added to the supporting information for Figures 3-5.

Reviewer 3

R 3-0) Recommendation: Publish in ACS Central Science after minor revisions noted.

Comments:
I enjoyed reading this paper. The effort to provide black box MR methods for organic reactivity is commendable and forward thinking. The paper is also well-written, well-structured and of the right length. I believe that it will create a fair bit of interest in the community.
I do not intend to nitpick on every single detail of the study. There is, however, one aspect that I feel uncomfortable with: the reference data is created by CCSD(T)/cc-pVDZ(page 8). It is well-known, that CC and DFT converge to the basis set limit at very different rates. Since cc-pVDZ is so very far from the basis set limit, I would argue that its use to generate reference data is nearly meaningless. The deviations from the basis set limit will be pretty much as large or larger than the deviations between different methods. CCSD(T)-F12/cc-pVDZ-F12 would do a much better job or any of the available local CC methods properly CBS extrapolated.
While redoing all of the reference data may not be practical, I think the authors should take at least a subset of small systems and generate proper CBS reference data that they then can compare their APC results to. Readers should know how these deviations compare to the ones one is getting using the more approximate reference data. One system would not be enough to convince me.
But apart from that - very interesting study that I definitely support.

We thank the reviewer for their positive review of this work. The point about basis set performance across methods is acknowledged and is addressed and discussed in response to R1-3.

In addition to reviewer comments, we have made the following changes in response to editorial comments:

Updated the abstract to fit the word limit as follows:

“In organic reactivity studies, quantum chemical calculations play a pivotal role ~~in serving~~ as the foundation of understanding and machine learning model development. While prevalent black-box methods like density functional theory (DFT) and coupled-cluster theory (e.g., CCSD(T)) have significantly advanced our understanding of chemical reactivity, they frequently fall short in accurately describing multiconfigurational transition states and intermediates. Achieving a more accurate description necessitates the use of multireference methods. However, these methods have not been used at scale due to their often-faulty predictions without expert input. Here, we overcome this deficiency ~~through the use of~~ with automated multiconfigurational pair-density functional theory (MC-PDFT) calculations. We apply this method to 908 automatically generated main-group organic reactions. We find ~~that~~ 68% of these reactions present significant multiconfigurational character, in which the automated multiconfigurational approach often provides a more accurate and/or efficient description than DFT and CCSD(T). This work presents the first high-throughput application of automated multiconfigurational methods to ~~chemical~~ reactivity, and is enabled by automated active space selection algorithms and the computation of electronic correlation with ~~the on-top functionals of~~ MC-PDFT on-top functionals. The combination of these two ~~new~~ developments can be used in a black-box fashion, avoiding significant active space inconsistency error in both single- and multireference cases and providing accurate multiconfigurational descriptions when needed.”
